# Quantitative Expression of Latent Disease Factors in Individuals Associated with Psychopathology Dimensions and Treatment Response

**DOI:** 10.1007/s12264-024-01224-z

**Published:** 2024-06-06

**Authors:** Shaoling Zhao, Qian Lv, Ge Zhang, Jiangtao Zhang, Heqiu Wang, Jianmin Zhang, Meiyun Wang, Zheng Wang

**Affiliations:** 1 Institute of Neuroscience, Center for Excellence in Brain Science and Intelligence Technology, State Key Laboratory of Neuroscience, Chinese Academy of Sciences, Shanghai, 200031 China; 2 University of Chinese Academy of Sciences, Beijing, 100101 China; 3 School of Psychological and Cognitive Sciences, Beijing Key Laboratory of Behavior and Mental Health; IDG/McGovern Institute for Brain Research; Peking-Tsinghua Center for Life Sciences, Peking University, Beijing, 100871 China; 4 Department of Medical Imaging, Henan Provincial People’s Hospital & the People’s Hospital of Zhengzhou University, Zhengzhou, 450003 China; 5 Tongde Hospital of Zhejiang Province (Zhejiang Mental Health Center), Zhejiang Office of Mental Health, Hangzhou, 310012 China

**Keywords:** Psychiatric comorbidity, Latent disease factor, Psychopathology dimension, Treatment outcome, Quantitative diagnosis

## Abstract

Psychiatric comorbidity is common in symptom-based diagnoses like autism spectrum disorder (ASD), attention/deficit hyper-activity disorder (ADHD), and obsessive-compulsive disorder (OCD). However, these co-occurring symptoms mediated by shared and/or distinct neural mechanisms are difficult to profile at the individual level. Capitalizing on unsupervised machine learning with a hierarchical Bayesian framework, we derived latent disease factors from resting-state functional connectivity data in a hybrid cohort of ASD and ADHD and delineated individual associations with dimensional symptoms based on canonical correlation analysis. Models based on the same factors generalized to previously unseen individuals in a subclinical cohort and one local OCD database with a subset of patients undergoing neurosurgical intervention. Four factors, identified as variably co-expressed in each patient, were significantly correlated with distinct symptom domains (*r* = –0.26–0.53, *P* < 0.05): behavioral regulation (Factor-1), communication (Factor-2), anxiety (Factor-3), adaptive behaviors (Factor-4). Moreover, we demonstrated Factor-1 expressed in patients with OCD and Factor-3 expressed in participants with anxiety, at the degree to which factor expression was significantly predictive of individual symptom scores (*r* = 0.18–0.5, *P* < 0.01). Importantly, peri-intervention changes in Factor-1 of OCD were associated with variable treatment outcomes (*r* = 0.39, *P* < 0.05). Our results indicate that these data-derived latent disease factors quantify individual factor expression to inform dimensional symptom and treatment outcomes across cohorts, which may promote quantitative psychiatric diagnosis and personalized intervention.

**Supplementary Information:**

The online version contains supplementary material available at 10.1007/s12264-024-01224-z.

## Introduction

Psychiatric heterogeneity and comorbidity are ubiquitous in the categorical diagnosis of mental disorders, which remains a bottleneck for precision diagnosis and individualized therapy [1–3]. Neurodevelopmental disorders such as autism spectrum disorder (ASD) and attention/deficit hyperactivity disorder (ADHD) not only demonstrated a high level of co-occurrence [4–6], but also bore a strong resemblance to clinical characteristics like inattention, social deficits, and anxiety [7–9]. Converging evidence suggests that these highly co-occurring symptomatic phenotypes may be mediated by common genetic risk factors [10–13] and brain dysconnectivity profiles across diagnoses [14–18]. This further extends to other psychiatric disorders like obsessive-compulsive disorder (OCD) which shared inhibitory control deficits and reduced function and structure in the rostral and dorsomedial prefrontal cortex with ASD [18, 19], and overlapping social deficits across OCD, ADHD, and ASD [20]. Together, these observations suggest that a single categorical assignment is not appropriate as the dimension of psychopathology may be represented as a distributed quantitative trait across diagnosis.

**Fig. 1:**
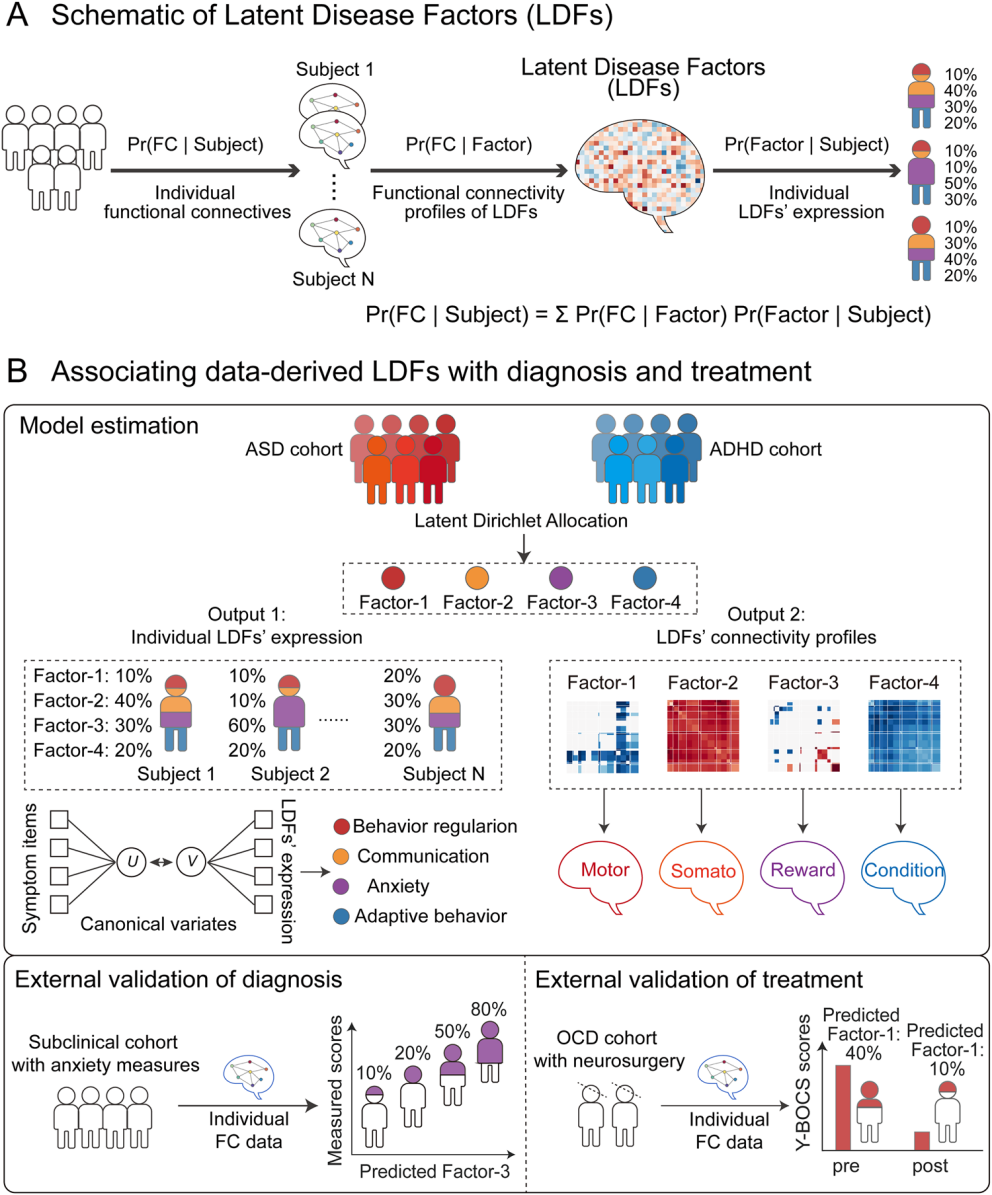
Identifying latent disease factors with a hierarchical Bayesian model. **A** Schematic of identifying latent disease factors (LDFs) with a hierarchical Bayesian model, which hypothesizes that each patient expresses a set of LDFs with varying probabilities ([Pr(Factor |Subject)]) and each LDF is characteristic of a specific functional connectivity (FC) profile ([Pr(FC |Factor)]). Thus, the Latent Dirichlet Allocation algorithm is applied to whole-brain FC data to unravel the connectivity profiles of LDFs and quantify their co-expression in each subject. **B** Flowchart of associating data-derived LDFs with psychopathology dimensions and treatment response across cohorts. LDFs that are derived from a hybrid cohort of patients with ASD or ADHD are subject to within-sample prediction of individual symptom scores by canonical correlation analysis. A meta-analytic tool – Neurosynth is also applied to associate cognitive functions with LDFs based on their connectivity profiles. As external validations, the estimated LDFs generalize to individuals from a subclinical cohort with anxiety measures and an OCD cohort with a subset of patients undergoing neurosurgical intervention to test whether these LDFs could be used to inform symptom severity and treatment outcomes in previously unseen individuals.

In contrast to a “winner-takes-all” assumption which assigns individuals exclusively to only one putative psychiatric category, the concept of quantitative diagnosis has been proposed based on the dimensional approach [21, 22], by which individuals are allowed to express more than one disease dimension/factor with varying degrees. Ideally in a four-disease-factor model, the factor composition of a patient might be 10% Factor-1, 40% Factor-2, 30% Factor-3, and 20% Factor-4 (Fig. 1A), with expression loading representing each factor’s likelihood. Thus, an individual patient is represented as a probability distribution on a fixed set of disease factors.

This biologically plausible statistical assumption has been methodologically tested in the context of neurodegenerative and neurodevelopmental diseases by adopting latent topic modeling techniques from natural language processing [23, 24]. They demonstrate that the estimated latent factors with distinct patterns of MRI-based brain features are likely to reflect the multi-faceted nature of individual patients who had mixed clinical profiles with impairments across multiple domains, thereby opening a new avenue to decompose clinical heterogeneity within disorders [25]. However, it remains unknown whether these latent factors could represent the psychopathology dimension of psychiatric comorbidity in different categorical disorders, which is a critical step toward fulfilling quantitative diagnosis for individual subjects [21, 26]. Furthermore, the diagnostic and predictive validity of this quantitative approach have not been investigated across categories and populations yet.

To address these questions, we built a hierarchical Bayesian model embedded with unsupervised latent Dirichlet allocation to estimate latent disease factors (LDFs) from whole-brain functional connectivity data in a hybrid cohort of ASD and ADHD (Fig. 1B). The model assumes that each subject expresses one or more LDFs with different probabilities, and each LDF is associated with a distinct functional connectivity (FC) profile. Associations between identified LDFs and psychopathology dimensions were evaluated with canonical correlation analysis (CCA) and a meta-analytic tool — Neurosynth, both of which help to pinpoint the neurobiological underpinnings of these data-derived factors. To assess the generalization performance, we examined whether LDFs estimated from the ASD+ADHD cohort were expressed in individuals from the Brain Genomics Superstruct Project (GSP) — a subclinical population with anxiety-related measures, and a local OCD cohort. Bayesian estimation was applied to predict factor expression in new individuals and repeated CCA analyses to investigate LDFs’ associations with symptom severity. Furthermore, we examined whether the predicted expression of LDFs could indicate treatment variability in a subset of OCD patients who underwent neurosurgical treatment. Models based on the same LDF generalized to previously unseen individuals and demonstrated accurate prediction of specific symptom domains, thus enabling the development of diagnostic and predictive network-level markers for quantitative diagnosis in individuals if validated in the prospective investigation.

## Materials and Methods

### Participants

We analyzed data from three publicly repositories: the Autism Brain Imaging Data Exchange II (ABIDE-II, fcon_1000.projects.nitrc.org/indi/abide/abide_II.html) [27], ADHD-200 (fcon_1000.projects.nitrc.org/indi/adhd200) [28], and the Brain Genomics Superstruct Project (GSP, neuroinformatics.harvard.edu/gsp) [29]. One composite OCD cohort from our local institutional database was also included [30–32].

For the ABIDE-II cohort, we included all patients with a diagnosis of either autism, Asperger syndrome, or pervasive developmental disorder not otherwise specified (PDD-NOS) according to the Diagnostic and Statistical Manual of Mental Disorders, Fourth Edition, Text Revision (DSM-IV-TR), as well as demographically matched healthy controls. For the ADHD cohort, we included all patients with ADHD measures including ADHD Index, Inattention, and Hyper/Impulsive, as well as demographically matched healthy controls. The participant inclusion criteria for these two sets were similar to the other ABIDE and ADHD-200 studies [28, 33], including a full IQ score higher than 80, known handedness, eye state information, and current medication status, images accepted after quality control, and meeting head motion criteria (e.g., a mean framewise displacement less than 0.2 mm). The resulting sample comprised 109 ASD patients (age = 11.7 ± 4.7; 95 males) with 173 healthy controls (age = 11.3 ± 3.8; 140 males) from ABIDE-II, and 106 ADHD patients (age = 11.5 ± 2.3; 88 males) with 131 healthy controls (age = 11.1 ± 2.0; 101 males) from ADHD-200, which were used as the discovery cohort for subsequent analyses. Additional information about the selection of the participants was provided in Fig. S1. Participants’ characteristics and data acquisition of this hybrid cohort were summarized in Table 1 (demographic information), Table S1 (imaging parameters), and Table S2 (behavioral scores). All datasets are anonymous and collected with the approval of the respective ethics boards.

**Table 1:** Demographic characteristics of participants in each data set

	ABIDE-II		ADHD-200		OCD		GSP
	ASD (*n =*109)	HC (*n =*173)	ADHD (*n =*106)	HC (*n =*131)	OCD (*n =*92)	HC (*n =*79)	GSP (*n =*502)
${\text{Age}}^{\text{a}}$, mean±SD	11.7±4.7	11.3±3.8	11.5±2.3	11.1±2.0	30.8±9.6	30.8±7.8	21.9±3.1
Gender^b^, number of M/F	95/14	140/33	88/18	101/30	56/36	50/29	218/284
FIQ, mean±SD	110±14.8	117±12.9	107± 13.1	117±13.1	–	–	114±8.6
Motion^c^, mean FD±SD	0.11±0.05	0.10±0.04	0.09±0.04	0.08±0.04	0.18±0.10	0.15±0.15	0.10±0.03
Medication, taken (%)	37, 33.9%	7, 4.1%	32, 30.2%	4, 3.0%	89, 96.8%	–	–
Handedness, right (%)	85.3%	89.0%	97.2%	95.4%	97.8%	98.7%	100%

FD: framewise displacement.

FIQ: full-scale IQ.

Participants with clinical diagnoses (ASD, ADHD, OCD) and healthy controls (HC) were compared with either 2-sample t-tests (for continuous measures) or ${\chi }^{2}$ tests (for categorical measures).

^**a**^ASD in ABIDE-II versus HC in ABIDE-II, *P =* 0.41, ADHD in ADHD-200 versus HC in ADHD-200, *P =* 0.10, baseline OCD versus HC in local dataset, *P =* 0.88.

^**b**^ASD in ABIDE-II versus HC in ABIDE-II, *P=*0.17, ADHD in ADHD-200 versus HC in ADHD-200, *P =* 0.26, baseline OCD versus HC in local dataset, *P =* 0.74.

^**c**^ASD in ABIDE-II versus HC in ABIDE-II, *P=*0.06, ADHD in ADHD-200 versus HC in ADHD-200, *P =* 0.13, baseline OCD versus HC in local dataset, *P =* 0.14.

Regarding the validation data sets, one subclinical cohort was included in which participants were enrolled from the GSP repository whose self-report and behavioral data are available, including a battery of negative affect and anxiety-related assessments. After applying the inclusion criteria including race with white, not Hispanic, right hand, known current medication status, and images accepted after quality control and a mean framewise displacement less than 0.2 mm, the final set comprised 502 participants (age = 21.9 ± 3.1; 218 males). Participants’ characteristics and behavioral scores were summarized in Table 1 and Table S3. Data collection and sharing were approved by the Partners HealthCare Institutional Review Board and the Harvard University Committee on the Use of Human Subjects in Research. Demographic information for each site in ABIDE-II, ADHD-200, and GSP sets can be found in Table S7.

As another external validation, we used one composite OCD cohort which included two retrospective data sets: baseline and capsulotomy. Part of the data from this cohort has been published previously [30–32, 34–36]. Yale-Brown Obsessive-Compulsive Scale (Y-BOCS) including obsession and compulsion was used as the clinical measure of OCD symptom severity. The inclusion and exclusion criteria for this OCD cohort were the same as in our previous studies [30–32] and described in supplementary materials. The final baseline set consisted of 92 OCD patients (age = 30.8 ± 9.6; 56 males) and 79 healthy controls (age = 30.8 ± 7.8; 50 males). In this dataset, 27 refractory patients (age = 30.4 ± 7.1; 17 males) underwent neurosurgical treatment with complete postoperative MRI scanning and clinical assessment. Characteristics of the baseline set were summarized in Table 1. Complete clinical and demographic information of two data sets were listed in Table S4. Age, sex, and head motion were matched between patients and controls for the baseline set. More detailed information was described in supplementary materials. Note that all participants were anonymized and retrieved from electronic databases for data analysis. Because we only used data from previously published studies and all the data were fully anonymized, no institutional review board approval was sought, and patient consent was not obtained for this re-analysis study.

### MRI Data Preprocessing

For three public datasets of ABIDE-II, ADHD-200, and GSP, we adopted a minimally preprocessed volume version from the Preprocessed Connectomes Project (http://preprocessed-connectomes-project.org), including slice timing correction, motion correction, spatial normalization into Montreal Neurological Institute (MNI) space, resampled to 3 × 3 × 3 mm^3^ voxels and smoothing with a Gaussian kernel (full width at half maximum, FWHM = 6 mm). Friston-24 parameters of head motion, white matter, and ventricle signals were regressed out, followed by linear drift correction and temporal filtering (0.01–0.1 Hz). For the composite OCD cohort, the details of the scanning and preprocessing protocols were described in supplementary materials and our previous studies [30, 32].

### Functional Connectivity Network Construction

We organized the cortical parcellation with Yeo *et al.’s* cortical parcellation map [37], and the subcortical parcellation comprising ten regions (bilateral amygdala, caudate, putamen, pallidum, and thalamus) defined by the automated anatomical labeling atlas II (AAL-2) template [38] and four regions (bilateral hypothalamus and nucleus accumbens) defined by the FreeSurfer template [39]. This generated a whole-brain template with a total of 128 regions of interest, which constituted nine functional networks widely used in the resting-state fMRI literature, including the temporal parietal network, default mode network, control network, limbic network, salience/ventral attention network (comprising both the salience and cingulo-opercular networks), dorsal attention network, somatomotor network, and visual network, coupled with the subcortical structure. Pearson’s correlation coefficients between the mean time courses of any pair of regions were calculated to represent their functional connectivity, resulting in a 128 × 128 connectivity matrix. More details were included in supplementary materials.

### Latent Factor Modelling with a Hierarchical Bayesian Framework

We employed a hierarchical Bayesian model based on latent Dirichlet allocation to identify LDFs in a hybrid cohort of patients with ASD or ADHD. This method is originally developed to discover the latent topics in natural language processing [40]. The model assumes that each subject expresses one or more LDFs with different probabilities [Pr(Factor | Subject)], and each LDF is associated with a distinct functional connectivity (FC) profile [Pr(FC | Factor)]. Given the whole-brain FC data and a pre-defined number of factors, the model estimates the probability that a subject expresses a particular factor (factor expression) and the probability that the FC profile is associated with a specific factor (FC profile). Therefore, the model can uncover hidden relations from whole-brain FC data representing distinct patterns of dysconnectivity and quantify their co-expression within each subject. The parameters of FC profiles [Pr(FC | Factor)] in estimated LDFs were applied to the FC data of previously unseen individuals from validation data sets to predict the individual’s factor expression using Bayesian estimation (the variational inference algorithm). A bootstrapping procedure was also applied to estimate the confidence interval of the FC profile for each LDF. More details were described in supplementary materials.

### Post-hoc Analysis of Latent Disease Factors

To reveal the clinical relevance of LDFs, we adopted two approaches to evaluate the relations between identified LDFs and psychopathology dimensions. First, we applied CCA to find an optimal linear combination of clinical scores that maximally correlated with each LDF expression. Item-level scores from each clinical scale were input into CCA as clinical features, and we focused on those clinical scores that were significantly associated with LDF expression after false-discovery-rate (FDR) correction (*P* < 0.05). The structural coefficient for each clinical score was computed to interpret the relative importance in each CCA group. Age, sex, handedness, medication, head motion, FIQ, and sites were regressed out as covariates for ASD and ADHD groups. Age, sex, medication, head motion, disease duration, education, and sites were regressed out as covariates for the OCD group. Age, sex, education, head motion, FIQ, and sites were regressed out as covariates for the GSP group. Statistical significance was tested with 10,000 permutations accounting for different sites with FDR corrected for multiple comparisons, more details see supplementary materials. Data from ABIDE-II was collected from multiple sites, not all participants had the same behavioral measures. Therefore, we only included participants with complete measures for each behavior scale. For the ADHD-200 dataset, available clinical measures included ADHD index, Inattention, and Hyper/Impulsive. Available ASD or ADHD patients for each behavioral scale used in our analyses can be found in Table S2.

Second, we used a meta-analytic tool — Neurosynth (https://neurosynth.org/) to identify cognitive terms associated with each factor [41]. The connectivity profiles of LDFs were thresholded to retain the top 50% of FCs and used as inputs for Neurosynth. The noncognitive terms (e.g., anatomical and demographic terms) were removed and the top 20 items with the largest correlation coefficients were kept for each factor. A word-cloud plot in which the font size was scaled with their correlation coefficients generated by Neurosynth.

### Validations for Individual Diagnostic and Predictive Utilities

We performed a range of internal validations to ensure the stability and robustness of identified LDFs. First, we tested whether the number of LDFs was affected by confounding factors including age, sex, motion, site vs. age, sex, motion, site, hand, medication status, and FIQ (Table S6). A stable model tends to have a higher correlation between LDFs estimated with different regressors. Second, we tested whether the estimated LDFs were dependent on the brain template by using a different parcellation map — AAL-2 [38], and repeated the analyses (Fig. S3). The corresponding network IDs for each ROI in AAL-2 are listed in Table S5. Third, to evaluate the differences between factors derived from the hybrid cohort with factors derived from a single categoric sample, we compared factors derived from the hybrid cohort with factors derived from the ASD or ADHD group alone and their corresponding symptom dimensions (Fig. S4).

We next generalized these identified LDFs to individuals from multi-site validation datasets. We applied Bayesian estimation with the FC profiles of identified LDFs to predict factor expression of participants from the GSP cohort and the local OCD cohort with their own FC data and repeated CCA analyses on these two independent datasets that were identical to those performed on the ASD+ADHD cohort to evaluate associations between symptom scores with predicted loadings of factor expression. Furthermore, we also applied the estimated LDFs to a subset of OCD patients with capsulotomy and investigated whether the longitudinal changes in factor expression before and after neurosurgery could reflect the treatment outcomes of OCD patients. Due to the limited sample size and heterogeneity in treatment response, we used Spearman’s correlation to evaluate the relations between alterations in factor expression and symptom remission for these surgically treated OCD patients, with age, sex, medication, head motion, disease duration, education and sites regressed as covariates.

We further examined whether an individual participant was characteristic of dysconnectivity profiles that were represented by one or more LDFs identified above. We selected patients with extraordinary phenotypes (relatively larger or smaller symptom scores) who were likely to have a predominant expression of one LDF. We computed the Dice Coefficient to quantitatively evaluate the across-subject variance in the similarity of an individual’s connectivity profile with the FC profile of the expressed LDF. For the GSP and baseline OCD cohorts, the coefficient was defined as:

$$ \frac{{{\text{Number}} \;{\text{of}}\; 2 \times [\left( {{\text{top}} \,10\% \;{\text{weighted}}\;{\text{FC}}} \right) \cap \left( {{\text{FC}}\;{\text{profile}}\;{\text{of}}\;{\text{expressed}}\;{\text{LDF}}} \right)]}}{{{\text{Number}}\;{\text{of}}\,[\left( {{\text{top}}\;10\% \;{\text{ weighted}}\,{\text{FC}}} \right) + \left( {{\text{FC}}\;{\text{profile}}\;{\text{of}}\;{\text{expressed}}\;{\text{LDF}}} \right)]}} $$

The top 10% weighted FCs for GSP and baseline OCD cohorts were identified as the FCs with the largest connection strength. For the OCD cohort with neurosurgery, the top-weighted FCs were identified as the most altered FCs before and after neurosurgery with paired *t*-tests. The FC profile of the expressed LDF was the statistically significant functional connective identified with the bootstrapping procedure. The edge-wise threshold of the significance level was set at a *P* value of 0.05 and subject to the FDR correction for multiple comparisons.

### Statistics

We used the chi-square test and two-sample *t*-test to examine significant between-group differences in categorical and continuous variables reported in Table 1, respectively. The statistical significance of CCA was tested with 10,000 permutations accounting for different sites with FDR correction. Pearson correlation was used to assess the significance level of *post-hoc* correlation between expression loadings of LDF and clinical measures in different cohorts. An FDR-corrected *P* value < 0.05 indicated significant correlations. The longitudinal change of factor expression in OCD patients with neurosurgery was examined with a two-sided Wilcoxon rank sum test (*P* < 0.05), the altered FCs in those OCD patients were identified by a paired two-sample *t*-test and controlled the FDR for multiple comparisons.

## Results

### Latent Disease Factors (LDFs) Characterized with Distinct Connectivity Profiles

**Fig. 2:**
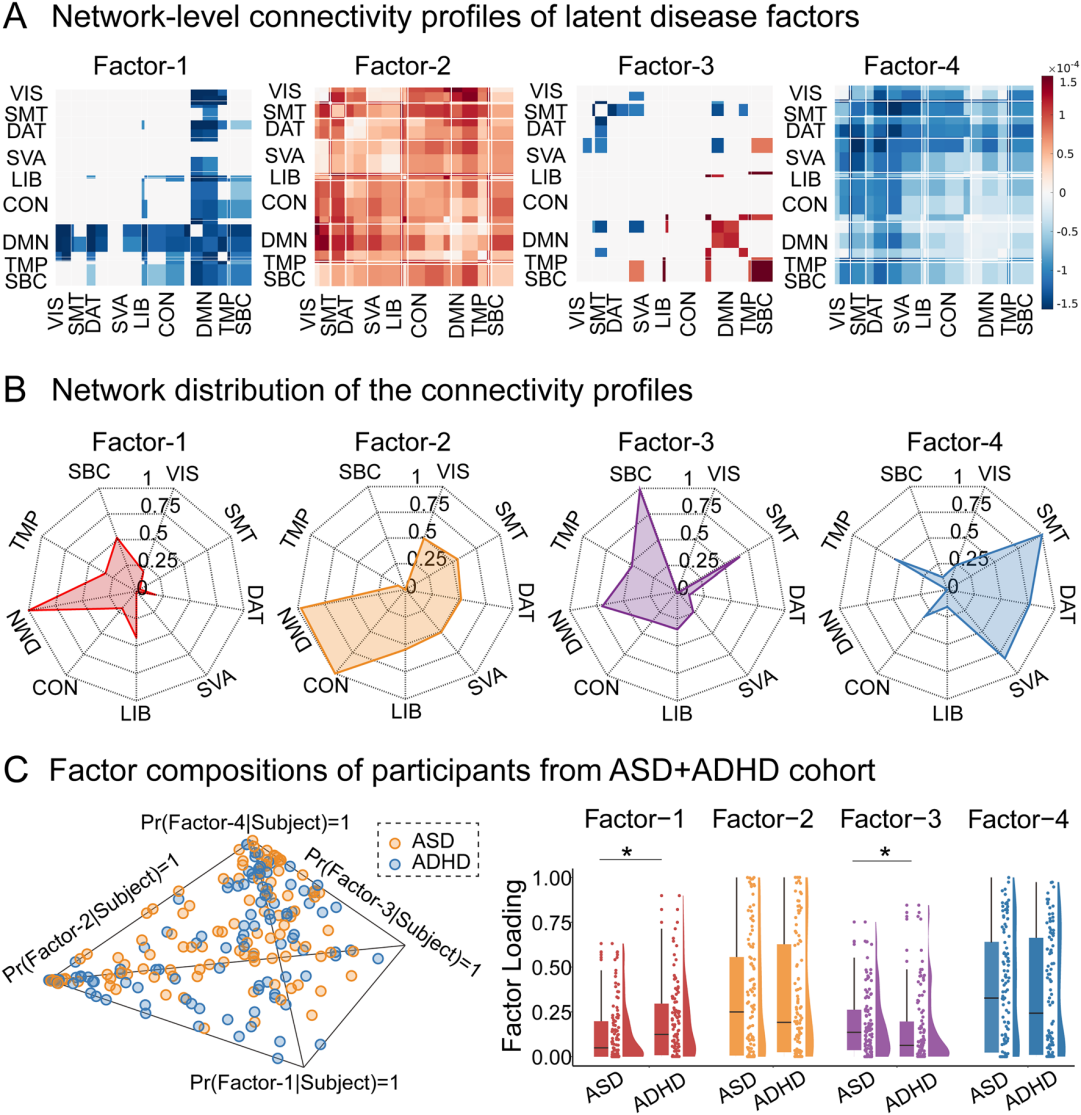
Latent disease factors estimated from a hybrid cohort of ASD and ADHD. **A** Network-level connectivity profile (Pr(FC | Factor)) of latent disease factors (LDFs), estimated from a hybrid cohort of ASD and ADHD and categorized into 9 functional networks, whereby only within- or between-network blocks with significant bootstrapped Z scores are selected (false discovery rate correction, *P* < 0.05, top 50% connectives are displayed). Warm colors indicate hyper-connectivity in the profile of LDFs relative to healthy controls whereas cool colors indicate hypo-connectivity. VIS: visual network; SMT: somatomotor network; DAT: dorsal attention network; SVA: salience/ventral attention network; LIB: limbic network; CON: control network; DMN: default mode network, TMP: temporal parietal network; SBC: subcortical network. **B** Network distribution of the connectivity profile for each LDF, thresholded and scaled to 0–1 to demonstrate its distribution on the network radar plot. **C** Factor compositions of 215 patients from the ASD+ADHD cohort. Each patient corresponds to a dot, with location representing the loadings of his/her factor expression. Corners of the tetrahedron represent pure factors, and dots closer to the corner indicate higher loadings of the respective factor. Graphs on the right panel compare the factor expression in ASD and ADHD, each dot represents a participant, and boxes denote the 25^th^ to 75^th^ percentile and the median line. Whiskers extend 1.5 times the interquartile range from the edges of the box. FDR correction was applied to account for multiple comparisons.

We first constructed a hybrid cohort of ASD and ADHD patients to derive LDFs with the hierarchical Bayesian model, which assembled data from ABIDE-II (*n* = 282) and ADHD-200 sample (*n* = 237). The model estimated statistically significant connectivity profiles for each LDF (Fig. 2A, the top 50% weighted connectives are displayed), and these connectivity features were quantified at the network level by assigning to the membership of 9 major networks (Fig. 2B). Thus Factor-1 largely overlapped with the default mode network (anterior temporal, dorsal, and media prefrontal cortex, etc., Fig. S5), whereas Factor-2 was mainly distributed in visual, somatomotor, default mode, and control networks. Factor-3 displayed a mixed pattern of hypo/hyper-connectivity in somatomotor, salience/ventral attention, and subcortical regions (hypothalamus, amygdala, etc., Fig. S5). Factor-4 was manifested as a marked decrease in somatomotor with dorsal attention and salience/ventral attention networks.

The probability that a subject expresses a particular factor was also estimated and we found each patient with ASD or ADHD co-expressed multiple factors with varying degrees, reflecting a continuum of individual variability (Fig. 2C). Moreover, significant group differences between ASD and ADHD were observed in Factor-1 and Factor-3 (two-sided Wilcoxon rank sum test, *P* = 0.034 for Factor-1, *P* = 0.034 for Factor-3, FDR corrected), in which ASD patients exhibited lower Factor-1 loading and higher Factor-3 loading compared with ADHD patients.

### Individualized Evaluation of Dimensional Phenotypes Based on LDFs Expression

**Fig. 3:**
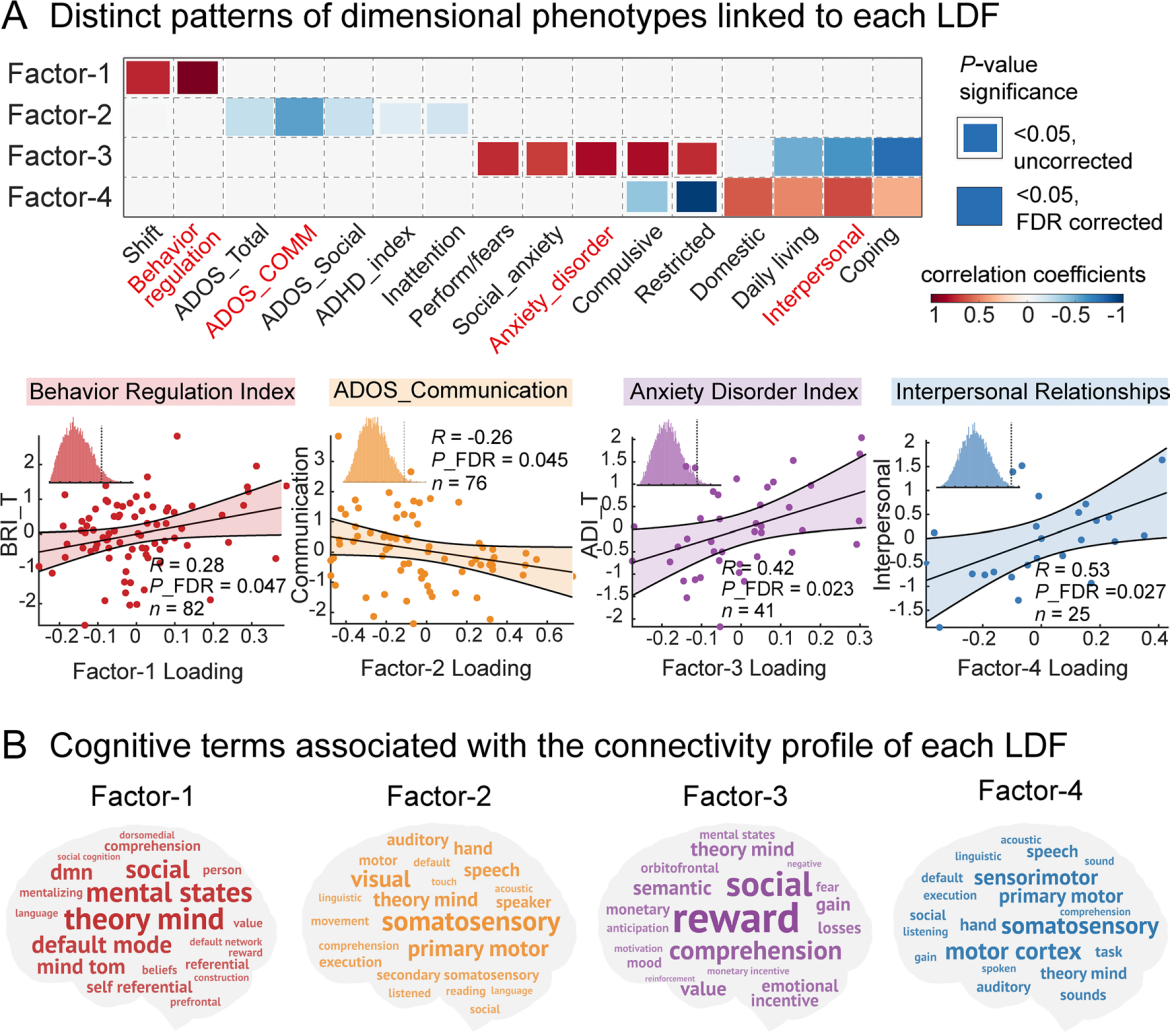
Mapping latent disease factors to distinct dimensional phenotypes. **A** Distinct pattern of dimensional phenotypes linked to latent disease factors (LDFs). The heatmap depicts the significant correlations between clinical scores and estimated LDFs (*P* < 0.05, FDR corrected). Symptoms with the largest correlation coefficients are labeled with red color and regarded as the representative clinical features for each LDF. The scatterplots show Pearson’s correlation between expression loadings of LDFs and the corresponding symptom scores, in which each dot represents a patient. Each insert displays the null distribution obtained by permutation testing (*n* = 10000), where dashed lines mark the actual correlation. **B** Cognitive terms associated with the connectivity profile of each LDF. Word clouds describe cognitive functions associated with each LDF. The connectivity profile of each LDF is the top 50% of functional connections over the whole brain as shown in Fig. 2A. In the word cloud plots, the font size of a given cognitive term corresponds to the correlation value with the meta-analytic map generated by Neurosynth (*r* = 0.065–0.3).

We adopted two approaches to evaluate the relations between identified LDFs and psychopathology dimensions. CCA analyses between factor expression and symptom scores revealed that Factor-1 was mainly associated with shift and behavioral regulation index in ASD, with the latter as the top contributing feature (*r* = 0.28, *P* = 0.047, FDR corrected, Fig. 3A). While Factor-2 corresponded to social communication domain in ASD patients (*r* = –0.26, *P* = 0.045, FDR corrected), it also correlated with ADHD-index and Inattention scores in the ADHD sample, although could not be corrected. Factor-3 was associated with anxiety-related symptoms in ASD including performance/fears, social anxiety, and anxiety disorder index (*r* = 0.42, *P* = 0.023, FDR corrected). Factor-4 corresponded to adaptive behaviors in ASD-like interpersonal relationships (*r* = 0.53, *P* = 0.027, FDR corrected). A meta-analysis based on Neurosynth indicated that the connectivity profile of Factor-1 primarily mapped onto default mode-related terms, while Factor-2 mainly mapped onto somatosensory processes, Factor-3 was strongly linked to reward, social, and emotions, and Factor-4 was largely associated with somatosensory and execution (Fig. 3B). In short, four identified LDFs were likely to represent distinct psychopathology dimensions of behavioral regulation, social communication, anxiety, and adaptive behaviors respectively.

**Fig. 4:**
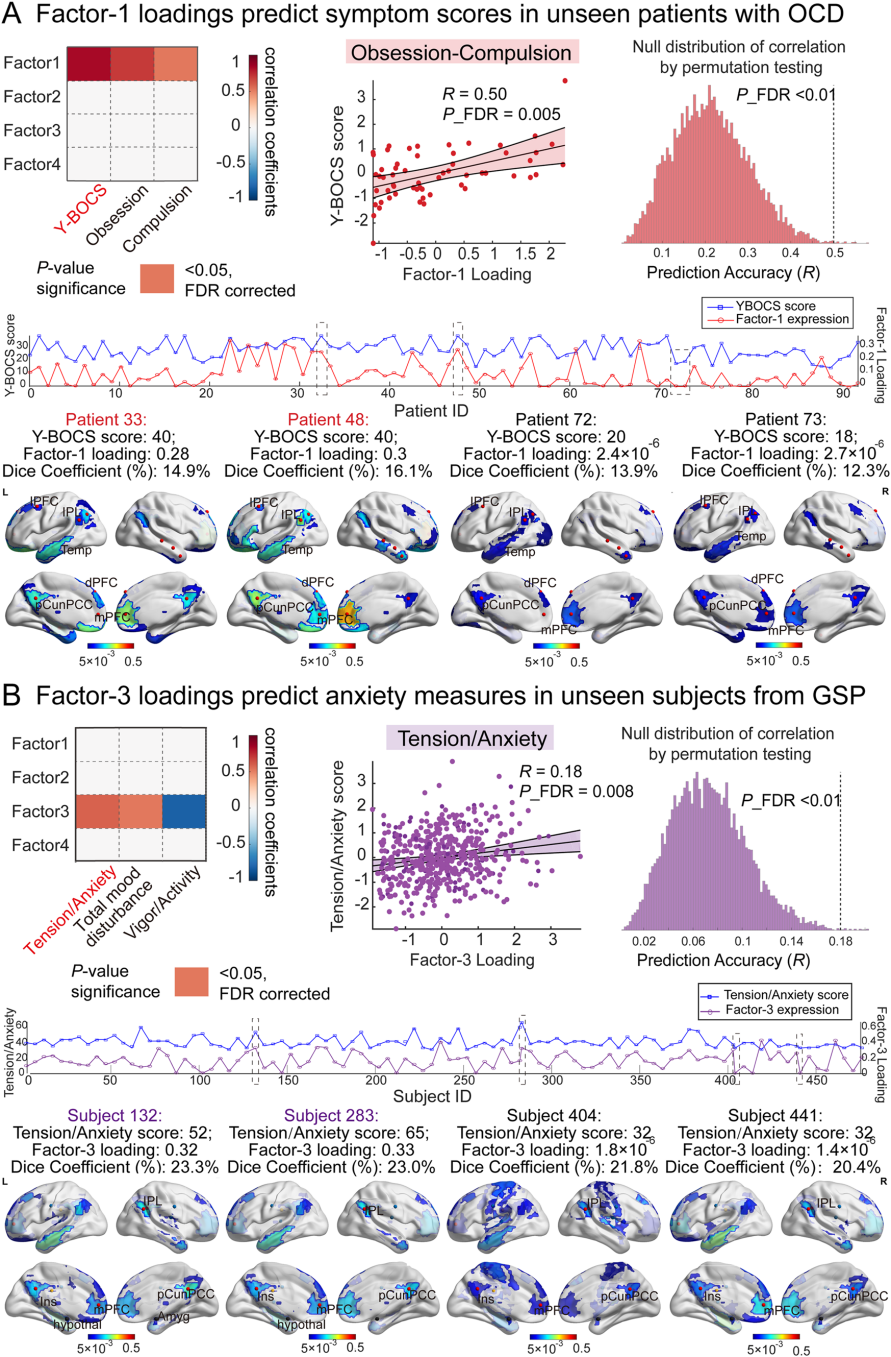
Individual factor expression informs symptom severity across cohorts. **A** The heatmap represents the correlation between predicted factor expressions with symptom scores from the Yale-Brown Obsessive-Compulsive Scale (Y-BOCS). The scatterplot describes the significant correlation between Factor-1 expression with measured Y-BOCS scores. The histogram provides evidence of stable prediction accuracy, which was far better than the null distribution of correlation by permutation testing. The dashed line marks the actual correlation score. The middle panel shows a one-to-one correspondence between the expression of Factor-1 and measured Y-BOCS scores for all 92 baseline OCD patients, in which four patients were selected for case demonstration. Patients with red ID had relatively larger Y-BOCS scores whereas the other two had relatively smaller scores. The bottom panel demonstrates the variably expressed connectivity profiles of four patients that overlapped with the connectivity profile of Factor-1. The color-coded areas on the brain surface map are the top contributing regions of the connectivity profile for each subject.** B** The heatmap indicates correlation coefficients between predicted factor expressions with symptom scores from the Proof of Mood States (PMOS) scale. The scatterplot shows significant correlations between expression loadings of Factor-3 with measured tension/anxiety scores from the PMOS scale. The histogram provides evidence of stable prediction accuracy, which was far better than the null distribution of correlation by permutation testing. The dashed line marks the actual correlation score. The middle panel shows a one-to-one correspondence between the expression of Factor-3 and tension/anxiety scores for all GSP participants whose PMOS scales were available, in which four participants were selected for case demonstration. Participants with purple ID had relatively larger clinical scores whereas the others had relatively smaller scores. The bottom panel demonstrates the variably expressed connectivity profiles of four subjects that overlapped with the connectivity profile of Factor 3. The color-coded areas on the brain surface map are the top contributing regions of the connectivity profile for each subject.

We further examined whether these identified LDFs generalized to previously unseen individuals from baseline OCD (*n* = 171) and GSP cohorts (*n* = 502) for quantitative diagnosis. We applied Bayesian estimation to predict the factor expression of participants from the external cohorts and calculated both the mean square error (MSE) and Pearson’s correlation between the predicted factor expression and clinical scores while controlling for confounding factors like age, sex, and motion. In OCD cohort, we found Factor-1 expressed in individuals at the degree to which the expression loadings were significantly correlated with Y-BOCS scores (*r* = 0.5, *P* = 0.005, FDR corrected, MSE = 0.011, Fig. 4A). Similarly, in the GSP cohort we observed a significant association between the expression loadings of Factor-3 with the tension/anxiety scores from the Profile of Mood States scale (*r* = 0.18, *P* = 0.008, FDR corrected, MSE = 0.004, Fig. 4B).

A case description was presented of four representative patients in the baseline OCD cohort who were determined by relatively higher or lower symptom scores. The ‘severe’ patients (Patient 33 and 48) had relatively higher Y-BOCS scores and Factor-1 expression (Y-BOCS score = 40, 40; Factor-1 loading = 0.28, 0.3, respectively, Fig. 4A). By contrast, the ‘mild’ patients (Patient 72 and 73) with relatively lower Y-BOCS scores had extremely minimum Factor-1 expression (Y-BOCS score = 20, 18; Factor-1 loading =${2.4\times10}^{-{6}}, \, {{2.7\times10}}^{-{6}}$, respectively). Two ‘severe’ patients also had larger Dice Coefficients than the ‘mild’ patients (14.9%, 16.1% vs. 13.9%, and 12.3%, respectively). The same analysis was also applied to subjects from the GSP cohort. The ‘high risk’ subjects (Subject 132 and 283) exhibited relatively higher anxiety levels and Factor-3 expression (tension/anxiety score = 52, 65; Factor-3 loading = 0.32, 0.33, respectively, Fig. 4B), while the ‘low risk’ subjects (Subject 404 and 441) had extremely lower expression in Factor-3 (tension/anxiety score=32, 32; Factor-3 loading =${{1.8} \, \times \, {10}}^{-{6}}, \, {{1.4} \, \times \, {10}}^{-{6}}$, respectively). And the ‘high-risk’ subjects also had larger Dice Coefficients than the ‘low-risk’ ones (23.3%, 23.0% *vs*. 21.8%, and 20.4%, respectively).

### Individualized Evaluation of Treatment Response Based on LDFs Expression

**Fig. 5:**
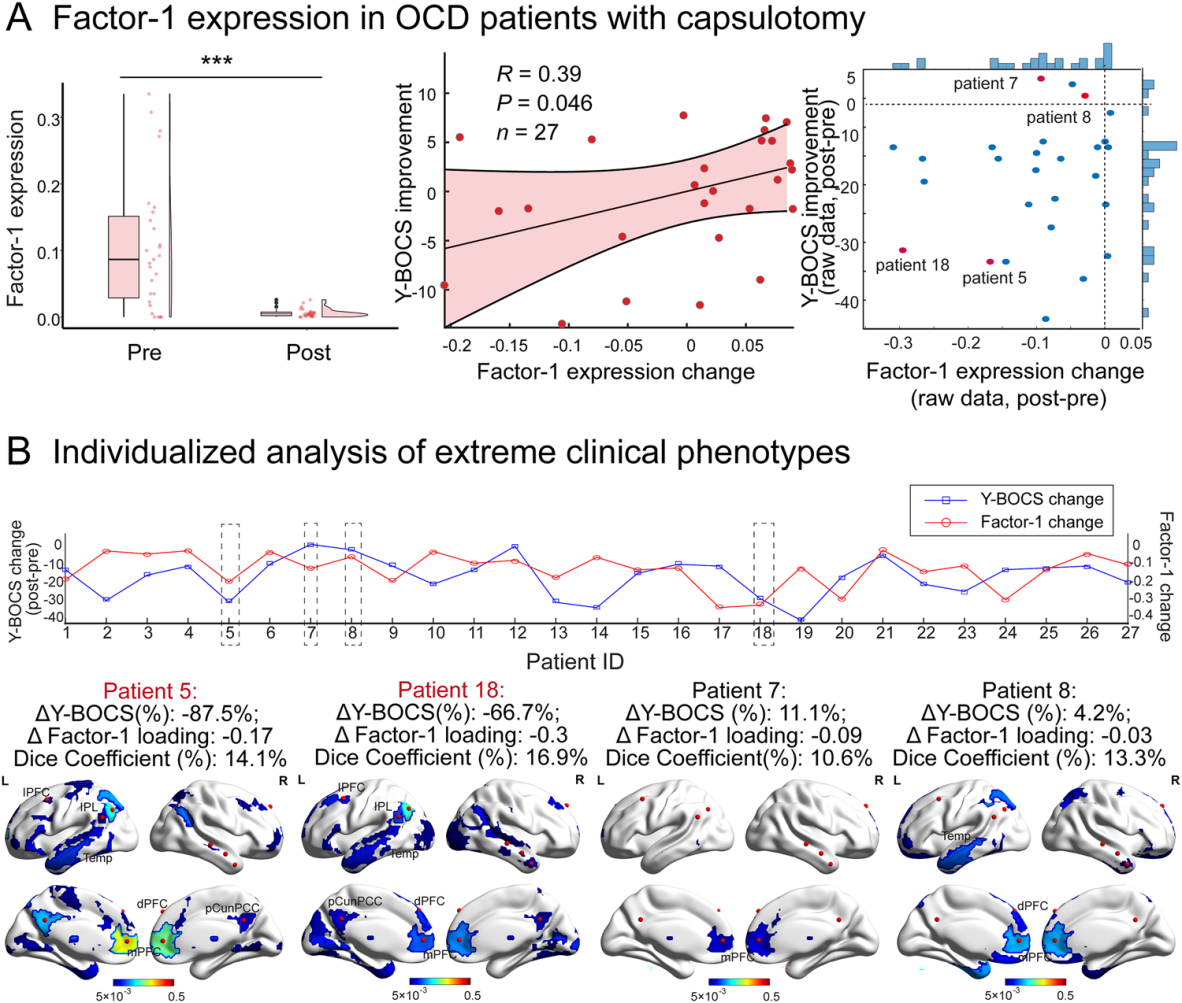
Individual factor expression informs treatment variability in OCD. A Factor-1 expression in OCD patients with capsulotomy informs treatment variability. We compared the expression loading of Factor-1 in each OCD patient before and after neurosurgery and found a significant decrease (***P < 0.001, two-sided rank sum test). The scatterplot shows the correlation between alterations in Factor-1 expression with measured Y-BOCS scores in those surgically treated patients. The right panel lays out the raw values of Factor-1 expression change and the Y-BOCS score improvement for each patient, in which Patients 5 and 18 are highlighted for pronounced symptom remission whereas Patients 7 and 8 are for poor treatment response. B Individualized analysis of extreme clinical phenotypes. The upper panel displays one-to-one correspondence between alterations in Factor-1 expression with measured Y-BOCS scores for all 27 treated OCD patients, in which four patients were selected for case demonstration, the patient’s ID with the red color indicated the relatively good treatment outcomes and the others were the relatively poor. The bottom panel illustrates the variably altered connectivity profiles of four patients that overlapped with the connectivity profile of Factor-1. The color-coded areas on the brain surface map are the top contributing regions of the altered connectivity profile for each subject.

We further asked whether quantitative expression of Factor-1 in patients with OCD could be used to inform treatment outcomes for those who underwent neurosurgical intervention. In a subset of 27 refractory OCD patients, we predicted factor expression of these individual patients before and after neurosurgery and found a significant decrease in expression of Factor-1 (two-sided Wilcoxon rank sum test, ${\text{P}} \, = \, {1.9\times10}^{-{7}}$, FDR corrected, Fig. 5A). More specifically, the reduction in Factor-1 expression was correlated with the decrease in Y-BOCS scores of these surgically treated patients (Spearman’s correlation, *r* = 0.39, *P* = 0.046, MSE = 0.017).

Furthermore, we included a case description of four representative patients who were determined by relatively good or poor responses to neurosurgical intervention. Patients with ‘good response’ (Patient 5 and 18) exhibited both pronounced symptom remission and larger decreases in Factor-1 expression after neurosurgery (Δ Y-BOCS = –87.5%, –66.7%, Δ Factor-1 loading = –0.17, –0.3, respectively, Fig. 5B). By contrast, patients with ‘poor response’ (Patient 7 and 8) presented even worse symptoms after neurosurgery and had minimal changes in Factor-1 expression (Δ Y-BOCS = 11.1%, 4.2%, Δ Factor-1 loading = –0.09, –0.03, respectively). Interestingly, alterations in the connectivity profile of OCD patients with ‘good response’ were substantially more similar to the FC profile of Factor-1 (the Dice Coefficient: 14.1%, 16.9%) than OCD patients with ‘poor response’ (the Dice Coefficient: 10.6%, 13.3%).

## Discussion

Utilizing a data-driven latent factor modeling approach, we identified a set of co-expressed LDFs by which individual variability in symptom severity and treatment efficacy were able to be quantified by loadings of LDFs in a Bayesian fashion across diagnostic categories and populations. Upon a closer look, each LDF was characteristic of distinct yet partially overlapping connectivity profiles. After within-sample validation, we observed the connectivity profile of Factor-1 primarily overlapping with the default mode network which has been previously reported to mediate behavioral inhibition and shift in ASD children [42, 43]. Factor 2 was related to social deficits in ASD children and inattention symptoms in ADHD children, consistent with previous findings that highlight links between inattention to social cognition problems in ADHD and ASD [44–46]. Its connectivity profile comprised networks involving not only higher-level cognitive functions but also sensorimotor processes, in accord with the current view of the core psychopathology of ASD and ADHD [47, 48]. In the meantime, Factor-3 was associated with multi-dimensional anxiety symptoms like fears and social anxiety, and its connectivity profile included connections between subcortical regions (especially the hypothalamus and amygdala) with the salience ventral attention network (e.g. insular), convergent with the insular–amygdala pathway previously reported in anxiety [49]. Factor 4 had most of the functional connections distributed between somatomotor and attention networks, which may implicate the pathophysiology of maladaptive behaviors [50, 51]. Collectively, these LDFs allowed us to uncover linked dimensions of psychopathology and dysconnectivity profiles that cut cross-diagnostic categories yet remain clinically interpretable.

### Latent Disease Factors Could Capture Stable Clinical Manifestations Across Diagnoses

We probed our study with external validations and found that LDFs could also help to indicate new individuals’ vulnerability to specific symptom impairment across diagnoses. External prediction revealed that the expression of Factor-1 which was associated with the behavioral regulation index in the ASD population was extended to inform obsessive and compulsive severity of individuals from an independent OCD cohort. The behavioral regulation index contains three subdomains: inhibition, shift, and emotion control, all of which reflect impairment of executive function in ASD [52–54]. Evidence from meta-analysis suggests that ASD and OCD both exhibit functional abnormalities in the dorsomedial prefrontal cortex underlying stereotyped and compulsive behaviors [19], which is one of the top contributing regions in the FC profile of Factor-1. In addition, deficits in inhibitory control are characteristic of ASD and OCD [55–58], intricately related to malfunction of the ventrolateral PFC and inferior parietal lobe, both of which are key regions in the FC profile of Factor-1.

### Shared Dysfunction with Subclinical Anxiety Was Characterized by Latent Disease Factors

The continuity hypothesis in psychiatry holds the view that clinically diagnosable disorders represent extreme expressions of traits that are distributed continuously within the general population [22], but it remains unclear how subclinical variation relates to the dimension of psychopathology. We found that except being associated with anxiety disorder index in ASD, Factor-3 whose profile included the amygdala, ventromedial prefrontal cortex, and insular was predictive of tension anxiety and negative emotion scores for individuals from the GSP cohort [59], indicating an anxiety-relevant circuit component shared between subclinical and clinically diagnosed populations [60, 61]. It was also predictive of the neuroticism scores in the GSP cohort, in accordance with the view that neuroticism serves as one of the major risk factors for anxiety [62]. This result supports the hypothesis that the psychopathology dimension of anxiety may lie on a continuous spectrum spanning healthy and diseased states [63–65], which merits further investigation of the underlying neurobiological mechanisms.

### Latent Disease Factors Could Facilitate a Personalized Treatment Evaluation

The last question we wanted to ask is whether LDFs could respond to symptom changes and capture sufficient individual differences between patients to provide a personalized treatment evaluation. We found that alterations of Factor-1 expression in patients with OCD were able to indicate symptom remission after neurosurgery, suggesting that the quantitative expression of LDF in individual patients is sensitive to capturing subtle differences in the brain circuits that may underlie treatment-induced symptom improvement. Illustrated in the case series of ‘good and poor responses’, the degree to which the connectivity profile of Factor-1 overlapped with peri-treatment changed functional connectives in patients would inform treatment variability in individuals. Future studies need to replicate the predictive utility of these data-derived LDFs for individual patients if validated in the prospective investigation, eventually predicting clinically relevant outcomes.

### Limitations of the Study

Our study has several limitations. First, participants in these four independent cohorts differed in some clinical characteristics including medication status, although demographic factors have been carefully controlled as covariates. Nevertheless, the questionnaire-based evaluation of symptomatic phenotypes in individuals was implemented with different scales across cohorts, which might cause biased measures. Future work is warranted to verify whether a single construct measurement and clinical confounds affect the quantitative expression of LDFs in individuals. Second, although Neurosynth is a valuable resource, it's essential to interpret its results cautiously and within the broader context of neuroscientific knowledge and empirical evidence. Third, we did not attempt to address the causal relationship between symptom severity and quantitative expression of LDFs in a cross-sectional study. It is crucial to design prospective studies to track longitudinally in individuals how the expression of LDFs evolves to impact the severity of core symptoms during disease progression, which is a stepping-stone to address the continuum of interindividual variability in psychiatry. Last, despite potential overlaps, we also admitted that caution is necessary for directly generalizing models or findings from one disorder to another due to the heterogeneity in neurobiological underpinnings of psychiatric disorders. In the future, studies specifically designed to compare computational models or neural features across ASD, ADHD, and OCD are essential to identify shared and disorder-specific neural signatures.

## Conclusions

Our findings collectively suggest that the latent disease factors based on the hierarchical Bayesian model capture multi-faceted relations between brain dysconnectivity and psychopathology dimensions, operate across different populations and diagnostic categories, and hold potential for individualized therapeutic evaluation, thus providing a promising approach for quantitative diagnosis and personalized intervention in psychiatry.

## Supplementary Information

Below is the link to the electronic supplementary material.

Supplementary file1 (PDF 1729 KB)

## Data Availability

This paper analyzes existing, publicly available data from ABIDE-II, ADHD-200, and GSP cohorts. The accession numbers for the datasets are listed above. The preprocessed OCD data has been deposited at Zenodo and is publicly available as of the date of publication. DOI: 10.5281/zenodo.8217579. Any additional information required to reanalyze the data reported in this paper is available from the lead contact upon request.

## Materials Availability

This study generated no new reagents or materials.
