# Supplementary material for: Quantitative Expression of Latent Disease Factors in Individuals Associated with Psychopathology Dimensions and Treatment Response
Source: Neurosci Bull. 2024 Jun 6;40(11):1667–80. doi: 10.1007/s12264-024-01224-z (PMC11607304; doi:10.1007/s12264-024-01224-z)
Supplement: Supplementary file 1 — Supplementary file1 (PDF 1729 KB) [file 12264_2024_1224_MOESM1_ESM.pdf]

Supplementary Materials and Methods

Participant Inclusion Criteria

ASD+ADHD Cohort

We used two publicly available repositories: the Autism Brain Imaging Data Exchange II (ABIDE-II) [1] and ADHD-200 sample [2] to construct a hybrid cohort of patients with autism spectrum disorder (ASD) and attention-deficit/hyperactivity disorder (ADHD). We chose ABIDE-II rather than ABIDE-I for its comprehensive phenotypic information and more balanced sex ratio. For this ASD cohort, we included all patients with a diagnosis of either autism, Asperger syndrome, or pervasive developmental disorder not otherwise specified (PDD-NOS), collectively referred to as the ASD group according to the Diagnostic and Statistical Manual of Mental Disorders, Fourth Edition, Text Revision (DSM-IV-TR), as well as demographically matched healthy controls. For the ADHD cohort, we included all patients with ADHD measures including ADHD Index, Inattention, and Hyper/Impulsive, as well as demographically matched healthy controls. The participant inclusion criteria for these two sets were similar to the other ABIDE and ADHD-200 studies [2, 3], including each acquisition site has at least 5 patients and 5 healthy controls (HCs), patients with a full-scale IQ score higher than 80; know handedness information, current medication status, and eye status at scan (open/closed); a mean framewise displacement (FD)[4] less than 0.2mm and the percent of frames or volumes with displacement greater than 0.2mm < 50%; MRI data with anatomical images providing near full brain coverage and successful registration. The resulting sample comprised 109 ASD patients with 173 matched HCs from ABIDE-II and 106 ADHD patients with 131 matched HCs from ADHD-200. The selection flowcharts of two datasets were listed in Fig. S1 and Fig. S2. Participants' characteristics and data acquisition of the hybrid ASD+ADHD cohort were summarized in Table 1, Table S1 (imaging

parameters), and Table S2 (behavioral scores). Demographic information for each site was summarized in Table S7.

GSP Cohort

The Brain Genomics Superstruct Project (GSP) is a multisite brain imaging repository constructed from a sample of non-clinically diagnosed individuals [5]. The publicly released GSP dataset consists of resting-state functional MRI and structural MRI scans of 1570 participants. Self-report and behavioral data are available for a subset of participants (n = 926). Inclusion criteria for participants in the GSP cohort included race with white not Hispanic, right hand, known current medication status, images accepted after quality control, and meeting head motion criteria (mean FD < 0.2 mm). The phenotypes of participants were evaluated with five self-report measures associated with the experience of negative affect and anxiety [6], including the State-Trait Anxiety Inventory [7], the NEO Five-factor of personality inventory [8], Behavioral Inhibition/Behavioral Activation Scale [9], Profile of Mood States scale [10], and Temperament and Character Inventory [11]. The resulting sample comprised 502 participants with 218 males. Participants' characteristics and behavior measures were summarized in Table 1 and Table S3.

OCD Cohort

The composite obsessive-compulsive disorder (OCD) cohort includes two retrospective data sets of OCD: baseline and capsulotomy. The baseline set consisted of 104 OCD patients and 82 HCs which were anonymously enrolled from our institutional database. Yale-Brown Obsessive-Compulsive Scale (Y-BOCS) was used as the clinical measure of OCD symptom severity. Among these 104 OCD

patients, 27 refractory patients underwent ventral capsulotomy with complete postoperative MRI scanning and clinical assessment. The inclusion and exclusion criteria for this OCD cohort were the same as in our previous studies [12–15], including the translation or rotation in any axis of head motion smaller than 3 mm or 3° during scanning; without any other neurological disorders, psychosurgery, current or past substance abuse or dependence, or pregnancy; and any relevant physical illness, such as brain tumor or brain injury, resulting 92 baseline OCD patients with 79 HCs, and 27 capsulotomy OCD patients. Age, sex, and head motion were matched between patients and controls for the baseline set. Part of the data from this OCD cohort has been published previously [12–16]. Complete clinical and demographic information of two data sets were listed in Table S4.

MRI Data Acquisition

The imaging parameters of ABIDE-II and ADHD-200 datasets are listed in Table S1. Participants in the GSP cohort were scanned in matched 3-T Siemens Tim Trio scanners with a 12-channel head coil. A T1-weighted image was collected for each subject with the following parameters: repetition time (TR) 2.2 s, echo time (TE) 1.5, 3.4, 5.2, and 7 ms, flip angle (FA) 7°, 144 sagittal slices width, 1.2 mm isotropic, duration 2min12s. One to two T2*-weighted gradient-echo echo-planar images (EPI) were collected for each subject with these parameters: TR 3s, TE 30 ms, FA 85°, 47 interleaved anterior commissure-posterior commissure aligned slices, 3 mm isotropic, duration 6 min 12 s. Participants were instructed to stay awake and keep their eyes open. More details on the MR data collection methods are available at https://www.neuroinfo.org/gsp/. Baseline and capsulotomy OCD data were collected using a Siemens Tim Trio 3T scanner (Erlangen, 5, Germany). Resting-state fMRI scans of the whole brain were acquired using a T2*-weighted EPI sequence: TR = 3000 ms; TE = 30 ms; FA =

90°; 47 axial slices; 3 mm slice thickness with no gap and 300 volumes. During the resting-state scan, participants were instructed to stay awake, with their eyes closed, and remain motionless. High-resolution T1-weighted images used a magnetization prepared rapid gradient echo sequence: TR = 2300 ms; TE = 3 ms; inversion time = 1000 ms; FA = 9°; $1 \times 1 \times 1$ mm^3 spatial resolution.

MRI

For the imaging data of ABIDE-II, ADHD-200, and GSP cohorts, we used a minimally preprocessed volume version from the Preprocessed Connectomes Project (http://preprocessed-connectomes-project.org/abide/index.html) [17]. The processing steps included slice timing correction, motion correction, spatial normalization into MNI space, reslicing to $3 \times 3 \times 3$ mm^3 voxels and smoothing with a Gaussian kernel (full width at half maximum, FWHM = 6 mm). Friston-24 parameters of head motion, white matter, and ventricle signals were regressed out, followed by linear drift correction and temporal filtering (0.01–0.1 Hz). For the imaging data of the composite OCD cohort, the preprocessing pipeline included slice timing correction, motion correction, spatial normalization into MNI space, resampling to $3 \times 3 \times 3$ mm^3, and spatial smoothing with a 2 mm kernel to reduce the effects caused by surgical lesions [12]. More details regarding how to deal with the issues of incomplete scanning in the capsulotomy dataset were described in our previous studies [12, 15].

Functional Connectivity Matrix Construction

We organized the whole brain parcellation with Yeo *et al.'s* [18] cortical parcellation map, and subcortical parcellation comprising ten regions (bilateral amygdala, caudate, putamen, pallidum, and thalamus) defined by the automated anatomical labeling atlas II (AAL-2) template [19] and 4 regions

(bilateral hypothalamus and nucleus accumbens) defined by the FreeSurfer template [20]. This generated a whole-brain template with a total of 128 regions of interest (ROI). Pearson's correlation was computed among the average time series of 128 brain ROIs, yielding a 128 × 128 connectivity matrix for each participant. Potential effects of age, sex, head motion (mean FD), and site differences were regressed from all participants' connectivity matrices using a linear model. Regression coefficients were estimated only from HCs to retain any disease-specific interactions with participants' characteristics (e.g., age, sex). The correlation matrix of patients was z-normalized with respect to the matrix of healthy controls. A z-score larger (or smaller) than zero for a given ROI pair would indicate hyper-connectivity (or hypo-connectivity) relative to HCs.

Latent Disease Factors Based on the Hierarchical Bayesian Model

We utilized the Latent Dirichlet Allocation (LDA) algorithm [21] to estimate latent disease factors in the ASD+ADHD hybrid cohort. It is a three-level hierarchical Bayesian model that was originally developed to discover latent topics from a corpus of text documents in natural language processing. The model assumes that each document is a collection of words associated with a subset of K latent topics and each topic is represented by a probability distribution over a dictionary of words. To extend LDA to resting-state functional connectivity (RSFC) data, we consider patients as documents, latent disease factors as topics, and functional connectives as dictionary words. The premise is that each subject expresses one or more latent disease factors with different probabilities [Pr(Factor | Subject)], and each factor is associated with a distinct but possibly overlapping functional connectivity (FC) profile [Pr(FC | Factor)]. In the LDA model, hyperparameters α and β are associated with the Dirichlet distributions, α controls the prior on the distribution of topics (latent factors) in documents

(participants), which can be treated as the probability that a participant expresses a specific factor, while β controls the prior on the distribution of words (functional connectivity, FC) in topics (latent factors), which is the probability that a latent factor is associated with a specific FC. As both hyper- and hypo-connectivity have been implicated in the psychopathology of ASD and ADHD [3, 22–26], we adopted the extension of the LDA model from Tang's paper [27], by adding an additional binary variable to indicate whether it was hyper- or hypo-connectivity for each FC. More details can be found in previous LDA papers [27, 28] and are briefly summarized here:

Given a cohort of subjects with RSFC data and a fixed number of latent disease factors K, LDA seeks to estimate the probability that a subject expresses a latent disease factor (θ, Pr(Factor | Subject)) and the probability that a latent disease factor is associated with an FC (β, Pr(FC | Factor)).

Suppose there are K latent disease factors and D subjects, then θ is a $D \times K$ matrix, and each row sums to one. θ_d is the d-th row of the θ matrix and the k-th element indicates the probability that the d-th subject expresses the k-th latent disease factor. Suppose there are V FCs in the brain, then β is a $K \times V$ matrix. β_k is the k-th row of the β matrix and the v-th element ($\beta_{k,v}$) indicates the probability that the k-th latent disease factor is associated with the v-th FC.

The LDA model assumes a prior (Dirichlet(α)) on the factor composition (θ_d) for each subject. Given the factor composition (θ_d), the total FCs (N_d) associated with the d-th subject, the d-th subject is generated as follows:

1. Choose $N \sim$ Poisson(ξ). (distribution of the FC)

2. Choose θ $\sim$ Dir(α). (distribution of the latent disease factor)

3. For each of the n-th FC $w_{d,n}$:

(a) Sample a latent disease factor $z_{d,n}$ with θ_d (d-th row of the θ matrix).

(b) Sample an FC $w_{d,n}$ from $p(w_{d,n}|z_{d,n}, \beta)$ (distribution specified in $z_{d,n}$-th row of the β matrix)

Given the z-normalized RSFC data of participants and a pre-defined number of latent disease factors K, a variational expectation-maximization (VEM) algorithm is used to estimate the probability that a subject is associated with a latent disease factor θ and the probability that an FC is associated with a latent disease factor β. A detailed derivation of the VEM algorithm for LDA is provided in Blei's paper [21]. The definition of the additional probability ρ indicates that an FC will exhibit hyper- or hypo- connection can be seen in the supplementary methods in the paper [27].

For each predefined number of latent disease factors *K*, the estimation procedure was repeated with 100 random initializations, resulting in 100 estimates. The final estimate was obtained by selecting the solution closest to the remaining 99 estimates, which has the highest average correlation with the remaining 99 estimates. To estimate the confidence interval of the FC profile for each latent disease factor, we applied a bootstrapping procedure within and between 17 subnetworks divided from 8 brain networks and subcortical regions, resulting in 18 × 18 matrices before computing bootstrapped z-scores. The z-scores were then calculated with the bootstrap-estimated standard deviation, converted to *P* values, and corrected with false discovery rate (FDR) correction ($P < 0.05$).

Estimation and Inference of the Hierarchical Bayesian Model

Given the whole brain RSFC data and a pre-defined number of latent disease factors K, we are going to estimate the probability that a subject expresses a latent disease factor (θ, Pr(Factor | Subject)) and the probability that a latent disease factor is associated with an FC (β, Pr(FC | Factor)). Similar to the original LDA paper [21], we utilized the variational expectation-maximization algorithm to estimate the parameters. The algorithm involves iterating between the variational E-step and M-step until

convergence. In the E-step, α, β are assumed fixed and the following variational parameters are updated by iterating the following two equations until convergence.

$$\phi_{d,n,k} \propto \beta_{k,v(nd)} exp\left(\psi(\gamma_{d,k}) - \psi\left(\sum_{i=1}^{K} \gamma_{d,i}\right)\right) \quad (1)$$

$$\gamma_{d,k} = \alpha + \sum_{n=1}^{N_d} \phi_{d,n,k} \quad (2)$$

where ϕ is the variational parameter for z, so we can interpret $\phi_{d,n,k}$ as the posterior probability that the n-th FC of the d-th subject belonged to the k-th latent disease factor, γ is the variational parameter for θ, so we can interpret $\gamma_{d,k}$ as the posterior probability of the d-th subject exhibiting the k-th latent disease factor, $v(n_d)$ indexes the n-th FC of the d-th subject corresponded to, ψ is the digamma function.

In the M-step, ϕ and γ are assumed fixed. The hyperparameter α is updated using the Newton-Raphson algorithms and β is updated as follows

$$\beta_{k,v} \propto \sum_{d=1}^{D} \sum_{n=1}^{N_d} \phi_{d,n,k} \, w_{d,n}^{v} \quad (3)$$

where $\beta_{k,v}$ is the probability that the v-th FC is associated with the k-th latent disease factor, $\phi_{d,n,k}$ is the posterior probability that the n-th FC of the d-th subject is associated with the k-th latent disease factor. And $w_{d,n}^{v}$ is the n-th FC in the d-th subject corresponded.

Suppose we are given a new subject, assuming that we have already estimated α, β, then we can simply apply the E-step (Eq. (1) and Eq. (2)) till convergence to estimate the factor composition of the new subject. This can be accomplished by applying the E-step with the following equation:

$$\gamma_{d,k} = \alpha + \sum_{n=1}^{N_d} \phi_{d,n,k} \quad (4)$$

Upon convergence, we can interpret $\gamma_{d,k}$ as the factor expression of the subject based on his or

her own FC data.

Canonical Correlation Analysis Between Latent Disease Factors and Clinical Symptoms

Canonical correlation analysis (CCA) is used to identify and measure the associations between two sets of variables. It determines a set of canonical variates, orthogonal linear combinations of the variables within each set that best explain the variability both within and between sets. Suppose the factor expressions [Pr(Factor |Subject)] in a group of patients are $x1$, $x2$, $x3$, and $x4$ respectively (with age, sex, head motion, handedness, medication, FIQ, and sites regressed). What we are interested in is how a set of clinical symptom variables relates to specific loadings of factor expression. We performed CCA between each factor expression loadings and each group of clinical symptom scores (regressing out factors including age, sex, head motion, handedness, medication, FIQ, and sites). To interpret the relative importance of clinical items in this canonical component, the structural coefficient for each symptom score was computed. Briefly, the canonical component yielded a linear combination of these items, resulting in one overall CCA loading per subject. The correlation coefficient between a specific item score and the overall CCA loading was computed across subjects to represent the structural coefficient, a larger positive correlation indicating a stronger association with a factor expression. The resulting structural coefficients were plotted in the heatmap plots in Fig. 3A and Fig. 4, and a permutation test was performed to evaluate statistical significance. The permutation procedure accounted for the participants coming from different sites, by restricting the permutations to within each site.

Supplementary Results

Validation with a Different Number of Latent Disease Factors

To explore the optimal number of latent disease factors, we computed the three-factor model and four-factor model with fewer regressors (age, sex, motion, site) and more regressors (age, sex, motion, site, hand, medication status, and FIQ), and compared their Pearson correlations (Table S7). A stable model tends to have a higher correlation between latent disease factors computed with different regressors. Estimated latent disease factors and correlation analysis results with clinical symptoms of the three-factor model are listed in Fig. S2.

Validation with a Different Brain Template

To demonstrate whether the choice of parcellation map causes any impact on the main findings, we replicated our analyses using a different brain template, AAL-2. To define the corresponding network modules with Yeo *et al.*'s parcellation map, network nodes obtained from the AAL-2 template were assigned to one of the seven functional modules: default mode, frontoparietal, ventral attention, dorsal attention, visual, sensorimotor, and limbic. Together with the subcortical region, consisting of 8 brain networks (except for the temporal-parietal network). The network assignment for nodes in AAL-2 is listed in Table S5. Estimated latent disease factors and correlation analysis results are listed in Fig. S3.

Validation with a Single Cohort

We compared the differences between latent disease factors derived from the hybrid ASD+ADHD cohort and a single disease categoric (ASD or ADHD cohort) (Fig. S4). Although we observed latent disease factors derived from the ASD cohort alone associated with externalizing behavioral problems in patients (e.g., activity, adaptive, and community), these latent disease factors failed to generalize to

independent datasets included in the present study. Meanwhile, no significant correlations were identified between the latent disease factors estimated from the ADHD cohort alone and any clinical scores (ADHD index, Inattention, Hyper/Impulsive) in patients.

References

1. di Martino A, O'Connor D, Chen B, Alaerts K, Anderson JS, Assaf M, *et al*. Enhancing studies of the connectome in autism using the autism brain imaging data exchange II. Sci Data 2017, 4: 170010.

2. Consortium HD2 00. The ADHD-200 consortium: A model to advance the translational potential of neuroimaging in clinical neuroscience. Front Syst Neurosci 2012, 6: 62.

3. Di Martino A, Yan CG, Li Q, Denio E, Castellanos FX, Alaerts K, *et al*. The autism brain imaging data exchange: Towards a large-scale evaluation of the intrinsic brain architecture in autism. Mol Psychiatry 2014, 19: 659–667.

4. Power JD, Barnes KA, Snyder AZ, Schlaggar BL, Petersen SE. Spurious but systematic correlations in functional connectivity MRI networks arise from subject motion. NeuroImage 2012, 59: 2142–2154.

5. Holmes AJ, Hollinshead MO, O'Keefe TM, Petrov VI, Fariello GR, Wald LL, *et al*. Brain Genomics Superstruct Project initial data release with structural, functional, and behavioral measures. Sci Data 2015, 2: 150031.

6. Holmes AJ, Lee PH, Hollinshead MO, Bakst L, Roffman JL, Smoller JW, *et al*. Individual differences in amygdala-medial prefrontal anatomy link negative affect, impaired social functioning, and polygenic depression risk. J Neurosci 2012, 32: 18087–18100.

7. Spielberger CD, Gorsuch RL, Lushene RE. Manual for the State-Trait Anxiety Inventory. 1970. (Corpus ID: 142620845, https://www.semanticscholar.org/paper/Manual-for-the-State-Trait-Anxiety-

Inventory-Spielberger-Gorsuch/e6d09d04fc8737094c193da471e2a50a809f77d4)

8. Costa PT, McCrae RR. Normal personality assessment in clinical practice: The NEO Personality Inventory. Psychol Assess 1992, 4: 5–13.

9. Carver CS, White TL. Behavioral inhibition, behavioral activation, and affective responses to impending reward and punishment: The BIS/BAS Scales. J Pers Soc Psychol 1994, 67: 319–333.

10. Mcnair DM, Lorr M, Droppleman LF. Manual for the Profile of Mood States. 1971. (Corpus ID: 67926195, https://www.semanticscholar.org/paper/Manual-for-the-Profile-of-Mood-States-Mcnair-Lorr/4d52b575f9055d62903bc0b829c1da475f5d571f)

11. Cloninger CR. A systematic method for clinical description and classification of personality variants. A proposal. Arch Gen Psychiatry 1987, 44: 573–588.

12. Yin D, Zhang C, Lv Q, Chen X, Zeljic K, Gong H, *et al*. Dissociable frontostriatal connectivity: Mechanism and predictor of the clinical efficacy of capsulotomy in obsessive-compulsive disorder. Biol Psychiatry 2018, 84: 926–936.

13. Lv Q, Lv Q, Yin D, Zhang C, Sun B, Voon V, *et al*. Neuroanatomical substrates and predictors of response to capsulotomy in intractable obsessive-compulsive disorder. Biol Psychiatry Cogn Neurosci Neuroimaging 2021, 6: 29–38.

14. Zhan Y, Wei J, Liang J, Xu X, He R, Robbins TW, *et al*. Diagnostic classification for human autism and obsessive-compulsive disorder based on machine learning from a primate genetic model. Am J Psychiatry 2021, 178: 65–76.

15. Chen X, Wang Z, Lv Q, Lv Q, van Wingen G, Fridgeirsson EA, *et al*. Common and differential connectivity profiles of deep brain stimulation and capsulotomy in refractory obsessive-compulsive disorder. Mol Psychiatry 2022, 27: 1020–1030.

16. Zhang C, Kim SG, Li J, Zhang Y, Lv Q, Zeljic K, *et al*. Anterior limb of the internal capsule tractography: Relationship with capsulotomy outcomes in obsessive-compulsive disorder. J Neurol Neurosurg Psychiatry 2021, 92: 637–644.

17. Yan CG, Zang YF. DPARSF: A MATLAB toolbox for "pipeline" data analysis of resting-state fMRI. Front Syst Neurosci 2010, 4: 13.

18. Yeo BT, Krienen FM, Sepulcre J, Sabuncu MR, Lashkari D, Hollinshead M, *et al*. The organization of the human cerebral cortex estimated by intrinsic functional connectivity. J Neurophysiol 2011, 106: 1125–1165.

19. Rolls ET, Joliot M, Tzourio-Mazoyer N. Implementation of a new parcellation of the orbitofrontal cortex in the automated anatomical labeling atlas. NeuroImage 2015, 122: 1–5.

20. Fischl B, Salat DH, Busa E, Albert M, Dieterich M, Haselgrove C, *et al*. Whole brain segmentation: Automated labeling of neuroanatomical structures in the human brain. Neuron 2002, 33: 341–355.

21. Blei DM, Ng AY, Jordan MI. Latent dirichlet allocation. J Mach Learn Res 2003, 3: 993–1022. (https://dl.acm.org/doi/10.5555/944919.944937)

22. Cheng W, Rolls ET, Gu H, Zhang J, Feng J. Autism: Reduced connectivity between cortical areas involved in face expression, theory of mind, and the sense of self. Brain 2015, 138: 1382–1393.

23. Cerliani L, Mennes M, Thomas RM, Di Martino A, Thioux M, Keysers C. Increased functional connectivity between subcortical and cortical resting-state networks in autism spectrum disorder. JAMA Psychiatry 2015, 72: 767–777.

24. Zaslavsky K, Zhang WB, McCready FP, Rodrigues DC, Deneault E, Loo C, *et al*. SHANK2 mutations associated with autism spectrum disorder cause hyperconnectivity of human neurons. Nat Neurosci 2019, 22: 556–564.

25. McCarthy H, Skokauskas N, Mulligan A, Donohoe G, Mullins D, Kelly J, *et al*. Attention network hypoconnectivity with default and affective network hyperconnectivity in adults diagnosed with attention-deficit/hyperactivity disorder in childhood. JAMA Psychiatry 2013, 70: 1329–1337.

26. Sripada C, Kessler D, Fang Y, Welsh RC, Prem Kumar K, Angstadt M. Disrupted network architecture of the resting brain in attention-deficit/hyperactivity disorder. Hum Brain Mapp 2014, 35: 4693–4705.

27. Tang S, Sun N, Floris DL, Zhang X, Di Martino A, Yeo BTT. Reconciling dimensional and categorical models of autism heterogeneity: A brain connectomics and behavioral study. Biol Psychiatry 2020, 87: 1071–1082.

28. Zhang X, Mormino EC, Sun N, Sperling RA, Sabuncu MR, Thomas Yeo BT, *et al*. Bayesian model reveals latent atrophy factors with dissociable cognitive trajectories in Alzheimer's disease. Proc Natl Acad Sci U S A 2016, 113: E6535–E6544.

Supplemental Tables

Table S1 Imaging parameters of participants in ABIDE-II and ADHD-200 cohorts

ABIDE-II cohort

Parameter	Site							
	GU_1	NYU_1	NYU_2	TCD_1	UCLA_1	KKI_1	KUL_3	USM_1
MRI Scanner	Siemens Trio	Siemens Allegra	Siemens Allegra	Philips Achieva	Siemens Magnetom TrioTim	Philips Achieva	Philips Achieva Ds	Siemens TrioTim
Field of view (mm)	192	192	192	240	192	256	200	220
Matrix	64 × 64	80 × 80	80 × 80	80 × 80	64 × 64	84 × 81	80 × 78	64 × 64
Number of slices	43	33	33	37	34	47	45	40
In-plane resolution (mm)	3.0 × 3.0	3.0 × 3.0	3.0 × 3.0	3.0 × 3.0	3.0 × 3.0	3.0 × 3.0	2.5 × 2.56	3.4 × 3.4

Slice thickness (mm)	2.5	3	3	3.2	4	3	2.7	3
Slice gap (mm)	0.5	0	0	0.3	0	0	0.4	0.3
TR (ms)	2000	2000	2000	2000	3000	2500	2500	2000
TE (ms)	30	15	15	27	28	30	30	28
Total scan time (mm:ss)	5:14	6:00	6:00	7:06	6:06	6:40	7:00	8:06
Flip angle	90	82	82	90	90	75	90	90

ADHD-cohort

Parameter	Site				
	KKI	OHSU	Peking_1	Peking_3	Peking_3
MRI Scanner	Siemens Trio	Siemens Magnetom TrioTim	Siemens Magnetom TrioTim	Siemens Magnetom TrioTim	Siemens Magnetom TrioTim
Field of view (mm)	256	240	200	200	200
Matrix	84 × 81	--	--	--	--

Number of slices	47	36	33	33	33
In-plane resolution (mm)	3.05 × 3.15	3.8 × 3.8	3.1 × 3.1	3.1 × 3.1	3.1 × 3.1
Slice thickness (mm)	3	3.8	3.5	3.5	3.5
Slice gap (mm)	0	--	--	--	--
TR (ms)	2500	2000	2000	2000	2000
TE (ms)	30	30	30	30	30
Total scan time (mm:ss)	6:40	3:32	8:06	8:06	8:06
Flip angle	75	90	90	90	90
Eyes during scan	Opened	Opened	Opened/Closed	Opened/Closed	Opened/Closed

Table S2 Behavioral data of participants in ABIDE-II and ADHD-200 cohorts

For each behavioral scale, only a subset of ASD participants is available.

Scale	Subscale	Mean (SD)
ABIDE-II		
Autism Diagnostic Observation Schedule Generic scores (ADOS_G, n = 76)	TOTAL	10.26 (3.61)
	COMM	2.89 (1.25)
	SOCIAL	6.99 (2.53)
	STEREO_BEHAV	1.64 (1.50)
Vineland Adaptive Behavior Scales 2nd Edition scores (VINELAND, n = 25)	RECEPTIVE	11.56 (2.53)
	DOMESTIC	12.56 (2.38)
	DAILY LIVING	89.64 (11.63)
	INTERPERSONAL	10.04 (2.99)
	COPING	12.76 (2.60)
	PLAY	13.16 (3.22)

Repetitive Behaviors Scale-Revised scores (RBSR, *n* = 47)	COMPULSIVE	3.57 (4.09)
	RESTRICTED	3.66 (2.77)
Multidimensional Anxiety Scale for Children scores (MASC, *n* = 41)	PP_T (Performance Fears)	50.34 (11.06)
	SOCIAL_TOTAL_T	52.56 (11.89)
	ADI_T (Anxiety Disorder Index)	53.63 (11.21)
Behavior Rating Inventory of Executive Function scores (BRIEF, *n* = 82)	SHIFT_T	67.28 (13.68)
	BRI_T (Behavioral Regulation Index)	64.51 (11.81)
Conners Parent Rating Scales (CPRS, *n* = 23)	OPP	59.26 (12.57)
	COG_INATT	65.17 (12.70)
	CONN_ADHD	64.96 (11.78)
	EMOT_LABILITY	55.09 (12.12)
	CONN_GI_TOTAL	62.57 (12.36)
	DSM_INATT	65.52 (12.03)

ADHD-200		
	ADHD index (n = 79)	55.44 (13.53)
ADHD-200 Phenotypic	Inattention (n = 99)	45.22 (22.74)
	Hyper/Impulsive (n = 99)	40.69 (25.49)

Table S3 Behavioral data of participants in GSP cohort

For the PMOS (Proof of Mood State) scale, only a subset of participants is available.

Scale	Subscale	Mean (SD)
State-trait anxiety inventory for adults (STAI, *n* = 502)	trait Anxiety	36.17 (8.93)
	state Anxiety	32.61 (9.43)
The NEO Five-factor model of personality (NEO, *n* = 502)	Neuroticism	19.70 (8.63)
	Extraversion	31.07 (6.41)
	Openness	31.98 (5.91)
	Agreeableness	32.17 (6.41)
	Conscientiousness	32.23 (6.97)
Profile of Mood States (PMOS, *n* = 479)	Total Mood Disturbance	15.23 (16.30)
	Tension Anxiety	39.62 (7.09)
	Depression Dejection	40.10 (7.05)
	Anger Hostility	44.20 (6.74)

	Vigour Activity	59.00 (8.77)
Behavioral inhibition (BIS) and behavioral activation (BAS) scale (*n* = 502)	Drive	11.06 (2.26)
	Fun	12.24 (2.10)
	Reward	17.34 (1.96)
	BIS	20.63 (3.54)
Temperament and Character Inventory (TCI-9) (*n* = 502)	Novelty	58.45 (8.82)
	Reward Dependence	69.29 (10.91)
	Harm Avoidance	52.07 (12.26)

Table S4 Demographic and clinical characteristics of composite OCD cohort

	Baseline OCD<br>(*n* = 92)	Baseline HC<br>(*n* = 79)	Capsulotomy OCD<br>(*n* = 27)
Age (years)[a]	30.8 ± 9.6	30.8 ± 7.8	30.4 ± 7.1
Sex (No. of male/female) [b]	56/36	50/29	17/10
Disease duration (year)	9.7 ± 6.2	-	10.2 ± 6.0
Y-BOCS	29.0 ± 6.8	-	-
Y-BOCS (pre-capsulotomy)	-	-	29.9 ± 6.8
Y-BOCS (post-capsulotomy) [c]	-	-	16.1 ± 9.6

Values are mean ± standard deviation.

Y-BOCS, Yale-Brown Obsessive Compulsive Scale.

[a] Local baseline OCD *vs* local HC, $P > 0.05$, two sample *t*-test.

[b] Local baseline OCD *vs* local HC, $P > 0.05$, chi-squared test.

[c] Pre-capsulotomy *vs* post-capsulotomy Y-BOCS score, $P < 0.001$, paired two sample *t*-test.

Table S5 Yeo Network ID for ROIs in AAL-2 Atlas

Anatomy in AAL-2	Yeo Network ID	Anatomy in AAL-2	Yeo Network ID
Precentral_L	Somatomotor	Calcarine_R	Visual
Precentral_R	Somatomotor	Cuneus_L	Visual
Frontal_Sup_2_L	DMN	Cuneus_R	Visual
Frontal_Sup_2_R	DMN	Lingual_L	Visual
Frontal_Mid_2_L	FrontalParietal	Lingual_R	Visual
Frontal_Mid_2_R	FrontalParietal	Occipital_Sup_L	Visual
Frontal_Inf_Oper_L	FrontalParietal	Occipital_Sup_R	Visual
Frontal_Inf_Oper_R	FrontalParietal	Occipital_Mid_L	Visual
Frontal_Inf_Tri_L	FrontalParietal	Occipital_Mid_R	Visual
Frontal_Inf_Tri_R	FrontalParietal	Occipital_Inf_L	Visual
Frontal_Inf_Orb_2_L	DMN	Occipital_Inf_R	Visual
Frontal_Inf_Orb_2_R	DMN	Fusiform_L	Visual

Rolandic_Oper_L	Somatomotor	Fusiform_R	Visual
Rolandic_Oper_R	Somatomotor	Postcentral_L	Somatomotor
Supp_Motor_Area_L	Somatomotor	Postcentral_R	Somatomotor
Supp_Motor_Area_R	Somatomotor	Parietal_Sup_L	Dorsal Attention
Olfactory_L	Limbic	Parietal_Sup_R	Dorsal Attention
Olfactory_R	Limbic	Parietal_Inf_L	Dorsal Attention
Frontal_Sup_Medial_L	DMN	Parietal_Inf_R	Dorsal Attention
Frontal_Sup_Medial_R	DMN	SupraMarginal_L	Ventral Attention
Frontal_Med_Orb_L	DMN	SupraMarginal_R	Ventral Attention
Frontal_Med_Orb_R	DMN	Angular_L	DMN
Rectus_L	Limbic	Angular_R	DMN
Rectus_R	Limbic	Precuneus_L	DMN
OFCmed_L	Limbic	Precuneus_R	DMN
OFCmed_R	Limbic	Paracentral_Lobule_L	Somatomotor

OFCant_L	Limbic	Paracentral_Lobule_R	Somatomotor
OFCant_R	Limbic	Caudate_L	Subcortical
OFCpost_L	Limbic	Caudate_R	Subcortical
OFCpost_R	Limbic	Putamen_L	Subcortical
OFClat_L	Limbic	Putamen_R	Subcortical
OFClat_R	Limbic	Pallidum_L	Subcortical
Insula_L	Ventral Attention	Pallidum_R	Subcortical
Insula_R	Ventral Attention	Thalamus_L	Subcortical
Cingulate_Ant_L	DMN	Thalamus_R	Subcortical
Cingulate_Ant_R	DMN	Heschl_L	Somatomotor
Cingulate_Mid_L	Ventral Attention	Heschl_R	Somatomotor
Cingulate_Mid_R	Ventral Attention	Temporal_Sup_L	Somatomotor
Cingulate_Post_L	DMN	Temporal_Sup_R	Somatomotor
Cingulate_Post_R	DMN	Temporal_Pole_Sup_L	Limbic

Hippocampus_L	Limbic	Temporal_Pole_Sup_R	Limbic
Hippocampus_R	Limbic	Temporal_Mid_L	DMN
ParaHippocampal_L	Limbic	Temporal_Mid_R	DMN
ParaHippocampal_R	Limbic	Temporal_Pole_Mid_L	Limbic
Amygdala_L	Limbic	Temporal_Pole_Mid_R	Limbic
Amygdala_R	Limbic	Temporal_Inf_L	DMN
Calcarine_L	Visual	Temporal_Inf_R	Dorsal Attention

Table S6 Correlations between Latent Factors with Different Confounders

We used different confounders in the functional network construction step to compare the stability with three- and four-factor models. Because the three-factor model produced inconsistent results between regressing with fewer confounders (age, sex, motion, site) and more confounders (age, sex, motion, site, hand, medication, FIQ), we focused on the four-factor model in our manuscript.

	Factor 1	Factor 2	Factor 3	Factor 4
Factors with more regressors, $k = 3$	0.3921	0.9267	0.6457	NA
Factors with more regressors, $k = 4$	0.8846	0.9485	0.7439	0.8202

Original factors were regressed with age, sex, and motion.

Table S7 Demographic information of each site in ABIDE-II, ADHD-200, and GSP cohort

ABIDE-II

Site	GU_1		KKI_1		NYU_1	
	ASD (*n* = 29)	HC (*n* = 41)	ASD (*n* = 31)	HC (*n* = 73)	ASD (*n* = 25)	HC (*n* = 26)
Age, mean ± SD	11.2 ± 1.1	10.5 ± 1.7	10.4 ± 1.4	10.5 ± 1.3	10.6 ± 6.8	9.7 ± 3.5
Gender, number of M/F	25/4	20/21	22/9	67/6	24/1	24/2
FIQ, mean ± SD	117.5 ± 14.9	120.4 ± 14.5	105.5 ± 12.2	114.9 ± 11.5	106.6 ± 15.2	117 ± 14.5
Motion, mean FD ± SD	0.10 ± 0.05	0.09 ± 0.04	0.13 ± 0.04	0.11 ± 0.04	0.10 ± 0.05	0.07 ± 0.04
Medication，taken (%)	16, 55.1%	6, 14.6%	10, 32.3%	0, 0%	5, 20%	0, 0%
Handedness, right (%)	23, 79.3%	39, 95.1%	29, 93.6%	59, 80.8%	18, 72.0%	25, 96.2%

Site	TCD_1		UCLA_1		USM_1	
	ASD<br>(*n* = 12)	HC<br>(*n* = 18)	ASD<br>(*n* = 9)	HC<br>(*n* = 11)	ASD<br>(*n* = 3)	HC<br>(*n* = 4)
Age (mean ± SD)	14.8 ± 3.5	16.3 ± 2.8	12.5 ± 2.1	9.7 ± 2.1	25.2 ± 12.4	26.6 ± 9.2
Gender (<br>number of M/F)	12/0	18/0	9/0	7/4	3/0	4/0
FIQ (mean ± SD)	113.9 ± 14.0	120.1 ± 10.6	105.4 ± 9.6	116.2 ± 14.5	111 ± 28	112.5 ± 11.4
Motion (mean FD ± SD)	0.11 ± 0.04	0.09 ± 0.04	0.09 ± 0.04	0.10 ± 0.05	0.09 ± 0.07	0.10 ± 0.03
Medication, taken (%)	0,<br>0%	0,<br>0%	5,<br>55.6%	1,<br>9.1%	1,<br>33.3%	0,<br>0%
Handedness,<br>right (%)	12,<br>100%	18,<br>100%	9,<br>100%	10,<br>90.9%	2,<br>66.7%	3,<br>75%

ADHD-200

Site	KKI		OUSH		Peking_1	

	ADHD<br>(*n* = 18)	HC<br>(*n* = 32)	ADHD<br>(*n* = 20)	HC<br>(*n* = 29)	ADHD<br>(*n* = 22)	HC<br>(*n* = 21)
Age, mean ± SD	10.6 ± 1.6	10.3 ± 1.3	9.1 ± 1.2	9.2 ± 1.3	11.5 ± 2.4	11.7 ± 1.6
Gender (<br>number of M/F)	9/9	23/9	15/5	13/16	18/4	17/4
FIQ (mean ± SD)	107.8 ± 14.1	110.3 ± 9.9	111.1 ± 11.9	120.4 ± 12.5	98.4 ± 12.0	121.0 ± 14.5
Motion (mean FD ± SD)	0.10 ± 0.03	0.10 ± 0.04	0.10 ± 0.05	0.08 ± 0.04	0.09 ± 0.04	0.07 ± 0.03
Medication, taken (%)	4,<br>22.2%	4,<br>12.5%	5,<br>25%	0,<br>0%	8,<br>36.4%	0,<br>0%
Handedness,<br>right (%)	16,<br>88.9%	27,<br>84.4%	20,<br>100%	29,<br>100%	22,<br>100%	20,<br>95.2%
Site	Peking_2		Peking_3			
	ADHD<br>(*n* = 29)	HC<br>(*n* = 27)	ADHD<br>(*n* = 17)	HC<br>(*n* = 22)		

Age (mean ± SD)	12.8 ± 1.8	11.5 ± 1.8	13.2 ± 1.3	13.4 ± 1.1
Gender (<br>number of M/F)	29/0	26/1	17/0	22/0
FIQ (mean ± SD)	111.1 ± 12.9	122.0 ± 12.8	104.1 ± 9.9	113.7 ± 13.0
Motion (mean FD ± SD)	0.09 ± 0.03	0.07 ± 0.03	0.09 ± 0.04	0.09 ± 0.04
Medication, taken (%)	15,<br>51.7%	0,<br>0%	0,<br>0%	0,<br>0%
Handedness,<br>right (%)	28,<br>96.6%	27,<br>100%	17,<br>100%	22,<br>100%

GSP

Site	A<br>(*n* = 15)	B<br>(*n* = 119)	C<br>(*n* = 3)	D<br>(*n* = 26)	E<br>(*n* = 339)
Age (mean ± SD)	26.5 ± 3.4	22.6 ± 3.1	23 ± 0	21.7 ± 2.6	21.4 ± 2.8

Gender (<br>number of M/F)	11/4	71/48	0/3	13/16	148/191
FIQ (mean ± SD)	112.6 ± 8.5	114.0 ± 8.8	107.7 ± 8.1	116.1 ± 8.4	113.5 ± 8.6
Motion<br>(mean ± SD)	0.10 ± 0.03	0.10 ± 0.04	0.07 ± 0.01	0.10 ± 0.04	0.10 ± 0.03
Education<br>(mean ± SD)	17.3 ± 1.8	15.2 ± 1.9	15.7 ± 0.6	14.3 ± 1.6	14.4 ± 1.9

Supplemental Figures and Figure Legends

ASD

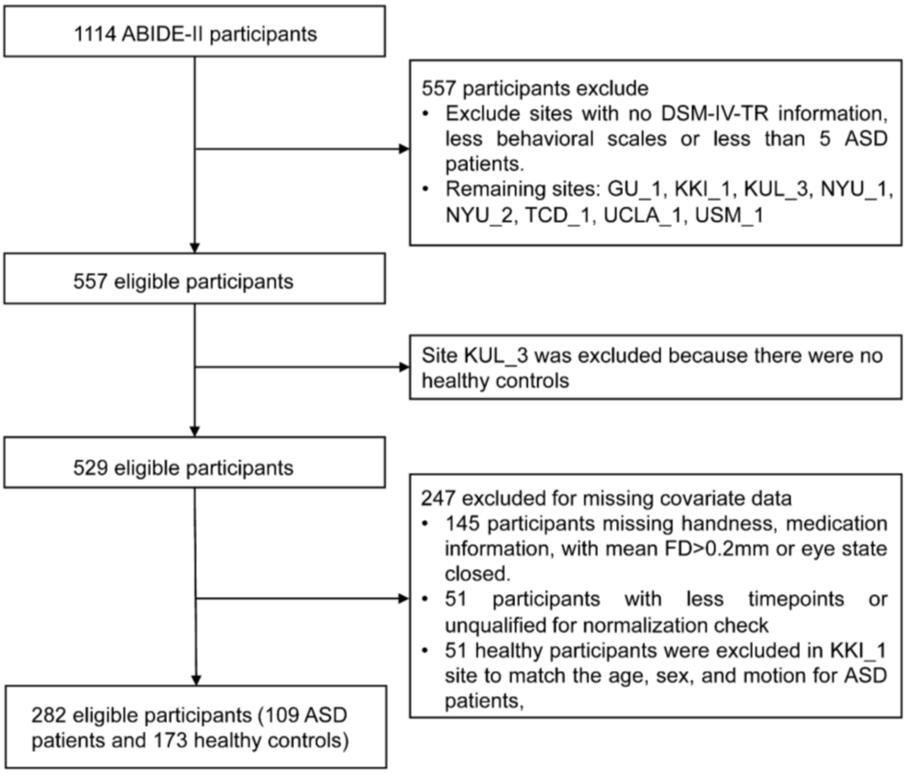

ADHD

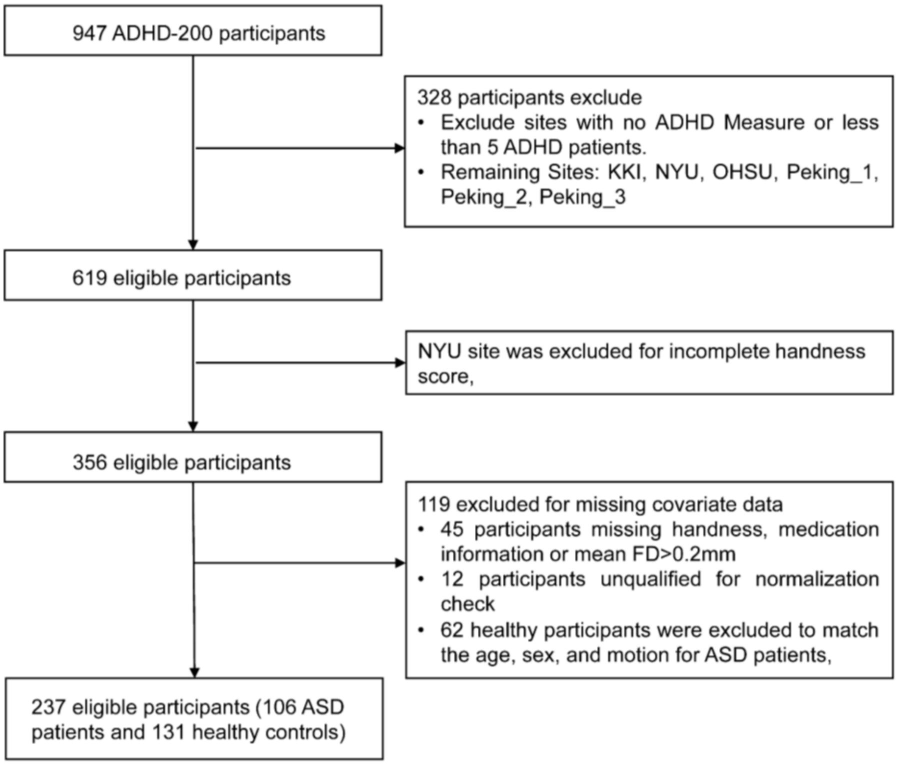

Fig. S1 Flowchart of participants selection in ABIDE-II dataset and ADHD-200.

DSM-IV-TR: The Diagnostic and Statistical Manual of Mental Disorders, Fourth Edition, Text Revision; Mean FD: mean framewise displacement; ADHD Measure: ADHD Index, Inattention and Hyper/Impulsive.

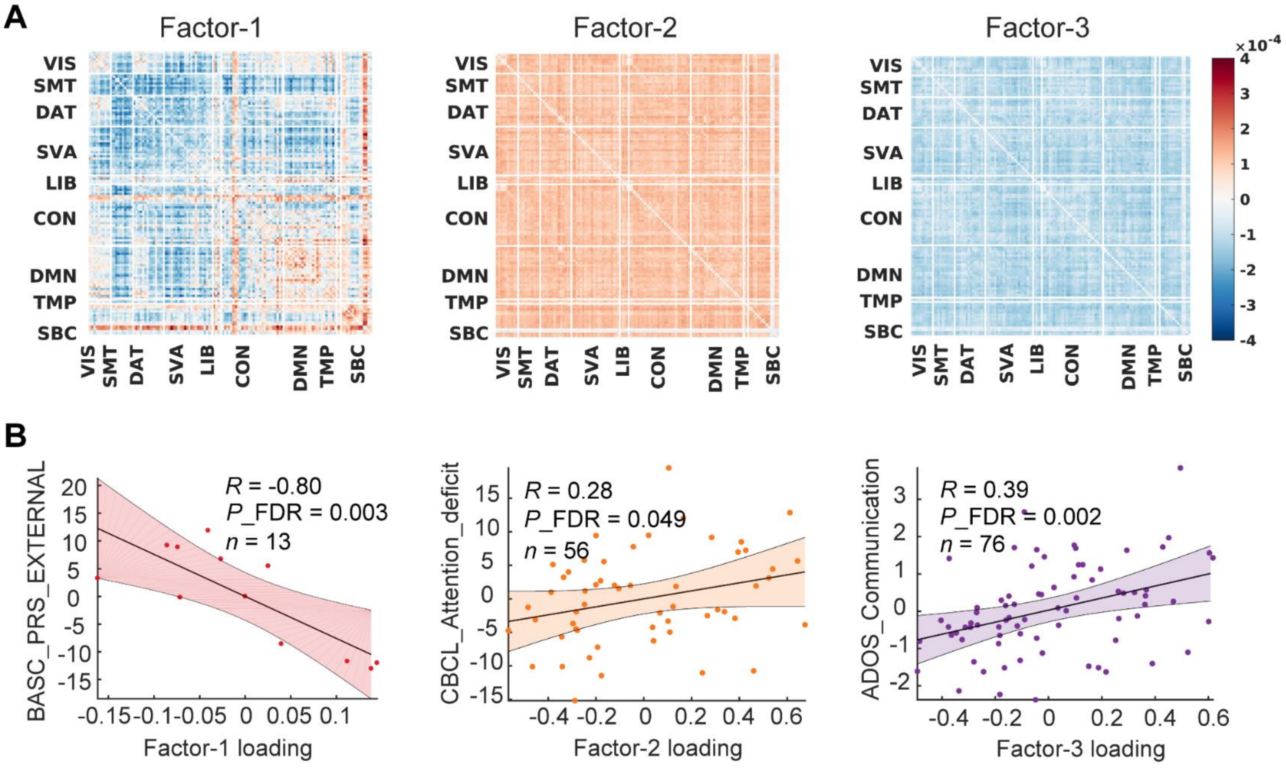

Fig. S2 Latent disease factors estimated with ASD+ADHD cohort (k = 3). **A** Connectivity profile (unthresholded) of latent disease factors (LDFs) estimated with the ASD+ADHD hybrid cohort (k = 3). Hot color indicates hyper-connectivity (relative to the healthy controls), and cold color indicates hypo-connectivity. **B** Correlation analysis results between factor expression loading and behavioral scores in LDFs estimated in the 3-factor model. Each dot in the scatter plot represents a patient and the p value is corrected with the FDR correction (P < 0.05). BASC_PRS_External: Externalizing Problems T Score from Behavior Assessment System for Children. CBCL_Attention_deficit: Attention Deficit/Hyperactivity Problems T Score from Child Behavior Checklist Ages 6–18. ADOS_Communication: Communication total score from Autism Diagnostic Observation Schedule Genetic.

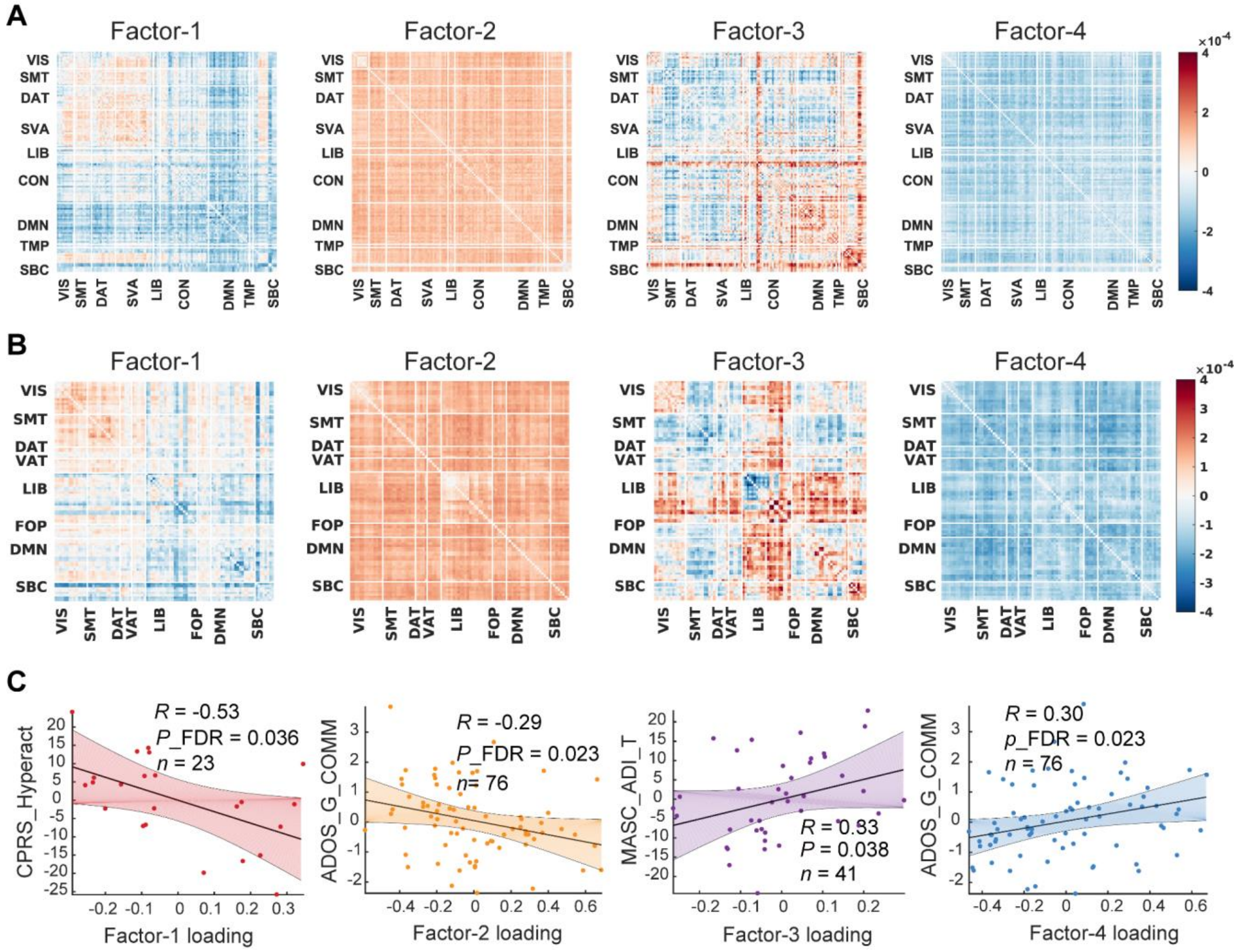

Fig. S3 Latent disease factors estimated with Yeo and AAL-2 template (k = 4). **A** Connectivity profile (unthresholded) of latent disease factors (LDFs) derived from the ASD+ADHD cohort, computed with Yeo's parcellation atlas. Hot color indicates hyper-connectivity (relative to the healthy controls), and cold color indicates hypo-connectivity. **B** Connectivity profiles (unthresholded) of LDFs derived from the ASD+ADHD cohort, computed with Automated anatomical labeling atlas II (AAL-2). **C** Pearson's correlation between factor expressions and behavioral scores in LDFs estimated with AAL-2 template. Each dot in the scatter plot represents a patient and the p value is corrected with the FDR correction ($P < 0.05$). CPRS_Hyperact: Hyperactivity score from Conners Parent Rating Scales. ADOS_G_COMM: Communication total score from Autism Diagnostic Observation Schedule Genetic. MASC_ADI_T: Anxiety Disorder Index T score from Multidimensional Anxiety Scale for Children.

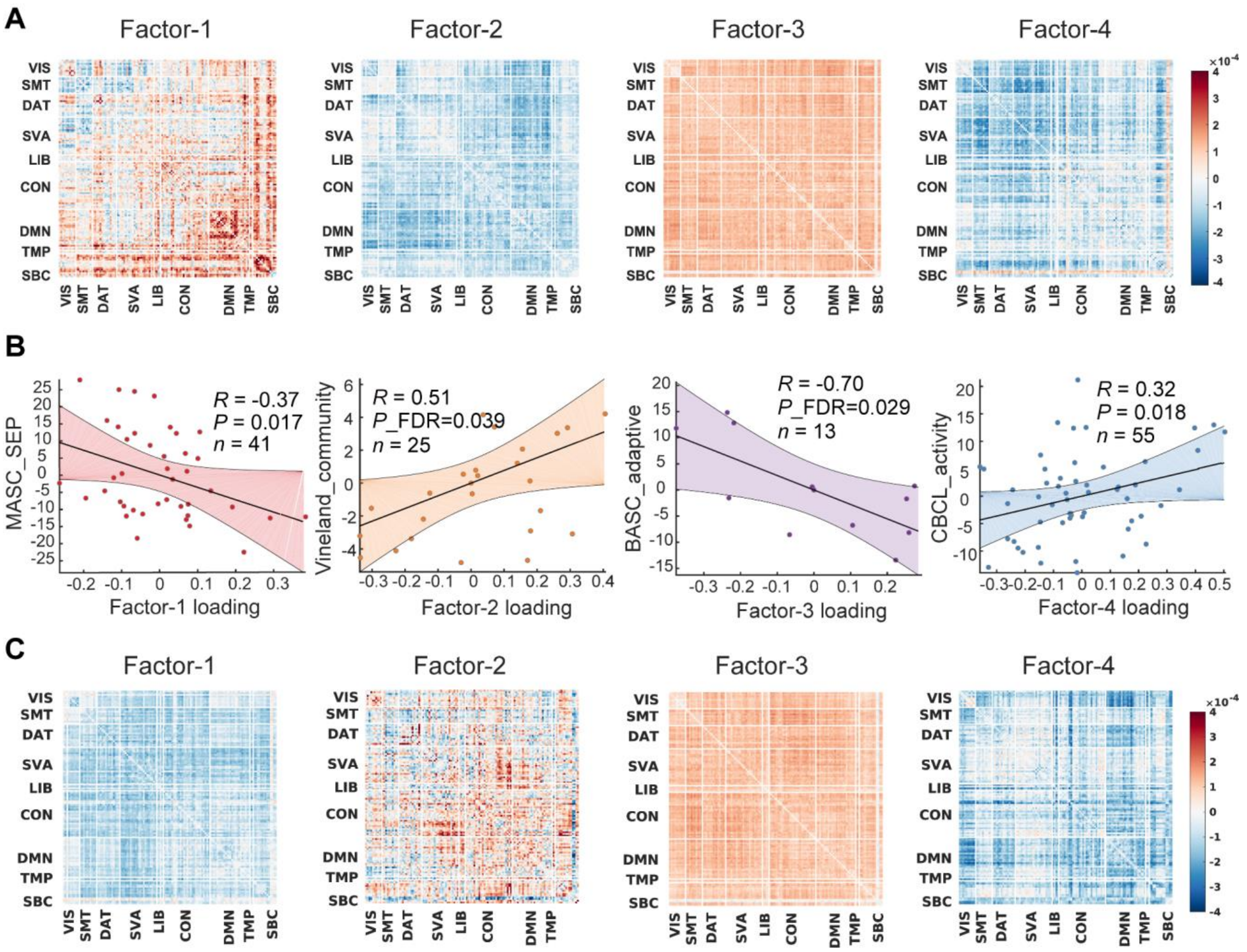

Fig. S4 Latent disease factors estimated with ASD or ADHD cohort (k = 4). **A** Connectivity profile (unthresholded) of latent disease factors (LDFs) estimated with ASD cohort. Hot color indicates hyper-connectivity (relative to the healthy controls), and cold color indicates hypo-connectivity. **B** Correlation analysis results between factor expression and behavioral scores in LDFs estimated with ASD cohort. Each dot in the scatter plot represents a patient and the P value is corrected with the FDR correction ($P < 0.05$). MASC_SEP: Separation/Panic score from Multidimensional Anxiety Scale for Children. Vineland_community: Community Daily Living Skills from Vineland Adaptive Behavior Scales. CBCL_activity: activities T score from Child Behavior Checklist. **C** Connectivity profiles (unthresholded) of LDFs estimated with ADHD cohort.

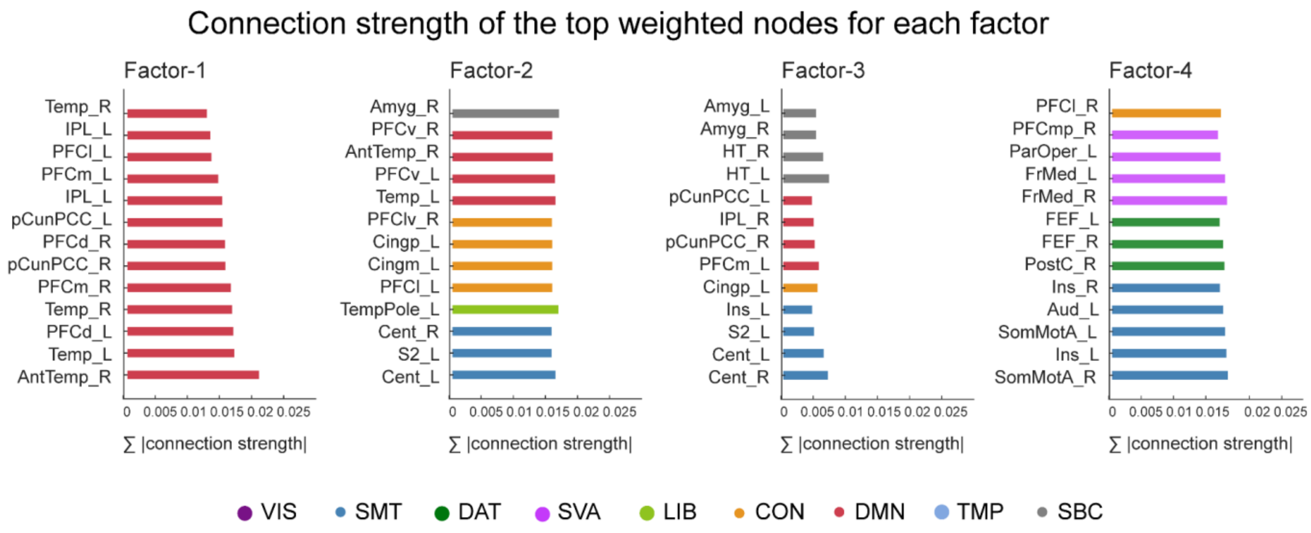

Fig. S5 Depicts of top contributing nodes based on the connection strength for each latent disease factor. Description of node-wise connection strength based on the estimated factor-specific connectivity profile, which is obtained by summing the absolute value of probabilistic weights of functional connectives connecting to each node.
